# Insertion of Telomeric Repeats in the Human and Horse Genomes: An Evolutionary Perspective

**DOI:** 10.3390/ijms21082838

**Published:** 2020-04-18

**Authors:** Marco Santagostino, Francesca M. Piras, Eleonora Cappelletti, Simone Del Giudice, Ornella Semino, Solomon G. Nergadze, Elena Giulotto

**Affiliations:** Department of Biology and Biotechnology, University of Pavia, 27100 Pavia, Italy; marco.santagostino@unipv.it (M.S.); mfrancesca.piras@unipv.it (F.M.P.); eleonora.cappelletti01@universitadipavia.it (E.C.); simone.delgiudice01@universitadipavia.it (S.D.G.); ornella.semino@unipv.it (O.S.); solomon.nergadze@unipv.it (S.G.N.)

**Keywords:** telomeres, telomerase, interstitial telomeres, insertion polymorphism, human genome, horse genome, evolution, DNA repair

## Abstract

Interstitial telomeric sequences (ITSs) are short stretches of telomeric-like repeats (TTAGGG)n at nonterminal chromosomal sites. We previously demonstrated that, in the genomes of primates and rodents, ITSs were inserted during the repair of DNA double-strand breaks. These conclusions were derived from sequence comparisons of ITS-containing loci and ITS-less orthologous loci in different species. To our knowledge, insertion polymorphism of ITSs, i.e., the presence of an ITS-containing allele and an ITS-less allele in the same species, has not been described. In this work, we carried out a genome-wide analysis of 2504 human genomic sequences retrieved from the 1000 Genomes Project and a PCR-based analysis of 209 human DNA samples. In spite of the large number of individual genomes analyzed we did not find any evidence of insertion polymorphism in the human population. On the contrary, the analysis of ITS loci in the genome of a single horse individual, the reference genome, allowed us to identify five heterozygous ITS loci, suggesting that insertion polymorphism of ITSs is an important source of genetic variability in this species. Finally, following a comparative sequence analysis of horse ITSs and of their orthologous empty loci in other Perissodactyla, we propose models for the mechanism of ITS insertion during the evolution of this order.

## 1. Introduction

Telomeres are nucleoprotein structures at the end of eukaryotic chromosomes. In vertebrates, telomeres are composed by extended arrays of the hexanucleotide TTAGGG [1] and by a specialized protein complex called shelterin [2]. The main role of telomeres is to prevent chromosome ends from being recognized and processed as double-strand breaks. In normal somatic cells, telomeres shorten at each replication round, while in germ-line and stem cells the reverse transcriptase telomerase ensures DNA replication of chromosome ends by adding TTAGGG repeats to the 3’ end of telomeric DNA. In normal somatic cells, after several replication rounds, telomeres reach a critical length, resulting in the loss of their ability to maintain genome stability. Short telomeres induce a state called replicative senescence, which is characterized by irreversible arrest of the cell cycle and is responsible for a decline in tissue renewal capacity [3,4,5,6]. Senescence can be seen as a barrier to uncontrolled cell proliferation and tumor development. Cells escaping senescence enter in a condition called crisis that is characterized by genome instability and ultimately leads to apoptosis. Rare survivor cells can rescue telomere maintenance mechanisms which can lead to cell immortalization and cancer. In addition, noncoding RNA molecules transcribed from telomeres (telomeric-repeat-containing RNA (TERRA)) participate in the regulation of telomere function [7,8] and have been proposed as prognostic markers in different types of tumors [9,10,11]. Therefore, telomeres together with telomerase, shelterin proteins and TERRA play crucial roles in the maintenance of genome integrity and stability.

Stretches of telomeric-like repeats are also located at internal sites [12]. Given their position, they are called interstitial telomeric sequences (ITSs). Whereas the role of telomeres in genome stability and replication is well-defined, the function of ITSs remains unclear.

ITSs can be classified according to their sequence organization and localization [13]. Het-ITSs are very extended blocks of telomeric-like repeats located at pericentromeric, terminal or intrachromosomal regions, generally coinciding with C-bands and easily detectable by cytogenetic analysis (fluorescence in situ hybridization (FISH)). They have been described in several vertebrate species [14,15,16,17,18,19] and in some insects [20] and plants [21] but are absent in other species such as human and mouse.

Although it has been proposed that Robertsonian fusion may be an important mechanism for ITS formation [13], in the human genome the only “fusion” ITS is the one on chromosome 2q13 whose repeated units are organized in a head-to-head fashion [22,23].

Some ITSs, that we called “subtelomeric” [13], are localized in regions immediately adjacent to the *bona fide* telomeres and contain many degenerate units intermingled with other types of tandem and interspersed repeats.

In the present work we focused on “short-ITSs” which are stretches of telomeric-like repeats ranging in size from a few to a few hundred repeat units that are distributed at internal chromosomal sites. Although short-ITSs have been studied only in a few species, we hypothesized that they may be present in all species in which telomeres are maintained by telomerase [13]. Short-ITSs, being too short to be efficiently visualized by FISH, can be found by sequence analysis of genomes. We previously studied the organization of short-ITSs in humans [23,24,25] and other primates [26,27,28] and in mouse and rat [25]. We also showed that different types of ITSs can coexist: short and het-ITSs in Chinese hamster [29,30]; short, subtelomeric and fusion-ITSs in humans [23]. In the horse, the absence of strong nonterminal telomeric FISH signals, that we now call het-ITSs, was first observed by de la Seña et al. [31]. With regards to other Perissodactyls, large, detectable by FISH, ITSs are also absent in the donkey [32] but present in five Hartmann’s Mountain zebra chromosomes [33].

Being composed by the repetition of 6 bp long units, short-ITSs are a particular type of microsatellite. Whereas canonical microsatellites originate from progressive expansion of a few pre-existing repeated units through DNA polymerase slippage [34], we showed that the mechanism of origin of ITSs during evolution is completely different [25,27]. Through a comparative analysis of human ITS loci and of their orthologous empty loci in other primates, we demonstrated that telomeric-like repeats appear suddenly during evolution, being introduced through a peculiar pathway of DNA double-strand break repair. We then proposed that telomerase may be directly involved in this pathway that should take place in the germ-line [25,27]. According to our model, the cytogenetic co-localization of short-ITSs and fragile sites that we previously observed in primates [26,28] and rodents [29,35] suggests they are not themselves prone to breakage but were rather inserted within DNA sites prone to breakage. Therefore, short-ITSs can be considered as ‘scars’ of DNA breaks that occurred at pre-existing fragile sites. 

Since ITS loci originated from the insertion of a telomeric repeat stretch at a DNA double-strand break site during evolution, we might expect that, for recently inserted ITS loci, empty ITS-less alleles are found in the same species, generating insertion polymorphism. Several studies demonstrated that transposable elements, such as Alu retrotransposons in humans, are characterized by insertion polymorphism [36,37,38,39,40,41,42]. This type of transposable element polymorphism can be involved in gene expression modulation leading to phenotypic consequences [43], including human and animal disease [44,45], and can be particularly informative for population genetics studies [46,47,48]. Interestingly, we showed that the insertion of an ERE1 retroelement within the promoter of the horse myostatin gene greatly reduces its expression and improves racing performance in some breeds [49]. We previously demonstrated that, in the horse, insertion polymorphism is particularly frequent for two types of sequences: (i) retrotransposons from the equine repetitive element 1 (ERE1) subfamily [49] and (ii) nuclear sequences of mitochondrial origin (numt) [50]. These observations, together with a number of molecular and cytogenetic comparative studies [51,52,53,54,55,56,57,58], support the hypothesis that the horse genome is in a stage of rapid evolution.

The first goal of the present work was to update the list of human ITSs and to investigate whether insertion polymorphism at ITS loci can be detected in the human population.

The second goal of this work was to identify ITS loci in the horse genome and to test whether, similarly to retrotransposons and numts, these loci are characterized by insertion polymorphism. 

The third goal of the present work was to study the molecular mechanisms of ITS insertion through sequence comparison between ITSs and their corresponding empty loci. 

## 2. Results

### 2.1. Identification of Human ITS Loci and Search of Insertion Polymorphism

We updated the list of human ITS loci by carrying out a BLAST search against the genome sequence assembly hg19/GRCh37. Using the sequence (TTAGGG)_4_ as query we identified 229 loci containing at least four telomeric repeats and less than one mismatch per unit, relative to the telomeric sequence. Supplementary Table S1 reports the complete list of human ITS loci together with their coordinates, length and number of mismatches.

We previously demonstrated that, unlike classical microsatellite repeats and similarly to other insertion sequences, ITSs arose from the sudden introduction of telomeric repeats into the genome. Since the fixation of a new genomic variant requires many generations, we expect that, at loci where ITS insertion occurred in evolutionarily recent times, the ITS-containing allele and the empty allele may be detected in the same population. 

In the attempt to identify empty alleles at human ITS loci, we took advantage of the 2504 genomes produced by the 1000 Genomes Project [59]. In this database, an ITS-less allele would miss part of the reference sequence (i.e., the telomeric repeat stretch), and therefore it would be classified as indel. We used the coordinates of the ITS loci listed in Supplementary Table S1 to manually test the presence of indels. We did not detect any deletion corresponding to ITSs, i.e., ITS-less alleles were not identified. Since the 4× genome coverage of this collection of human genomes would allow the detection of most variants with frequencies higher than 1%, we cannot exclude that rare ITS-less variants may be present in the human population. We would like to point out that, in the 1000 genomes database, all alleles at each variable locus are listed. Therefore, empty alleles could be detected both in heterozygous and in homozygous individuals. In other words, homozygosity is not necessary to detect empty alleles. On the other hand, if the reference genome was homozygous for an empty allele that was present as ITS in other individuals, with our approach we would not be able to detect it because this locus would not be included in our ITS list. However, since insertion polymorphism of human ITSs is extremely rare or even absent, we can reasonably suppose that this possibility is unlikely.

To test the presence of ITS-less alleles, we also carried out a PCR-based approach. For each locus, primer pairs were designed on the sequences flanking the telomeric repeat. We reasoned that empty alleles may only be present at ITS loci that were inserted recently in the human genome. We previously identified four ITSs that appeared in the human lineage after its separation from the chimpanzee lineage [25]. These ITSs are absent from all nonhuman primates and we called them “human-specific ITSs”. Using primer pairs flanking the four human-specific ITSs (Supplementary Table S2), we amplified genomic DNA of 209 individuals from different populations distributed worldwide. The results of this analysis are reported in Table 1. In accordance with the results of the in silico analysis, no ITS-less alleles were identified, confirming that insertion polymorphism of ITSs is either absent or very rare in the human population.

### 2.2. ITS Loci in the Horse Genome

**Figure 1:**
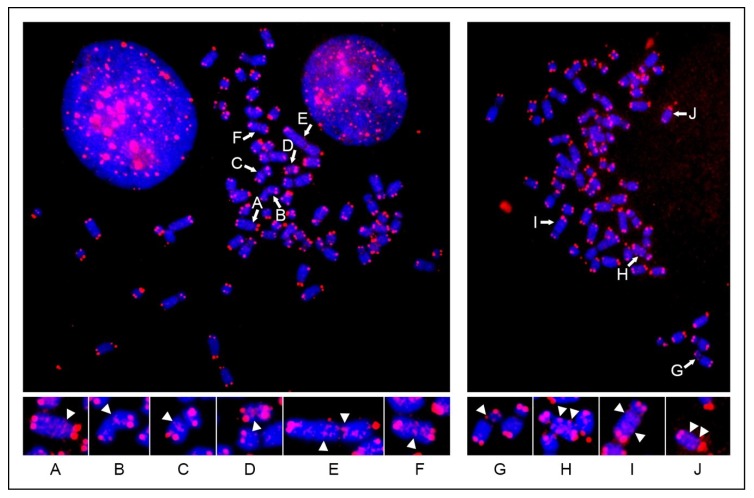
Fluorescence in situ hybridization with telomeric probe on two metaphase spreads from horse primary fibroblasts. Chromosomes are labelled with DAPI. Hybridization signals (red) at chromosome ends mark telomeres. Some of the chromosomes showing faint hybridization signals corresponding to short interstitial telomeric sequences are indicated (white arrows). Magnifications of selected chromosomes are reported below each metaphase, and some of the interstitial telomeric signals are marked by arrowheads.

We first performed FISH experiments on metaphase spreads of horse primary fibroblasts using the previously described telomeric repeat oligonucleotide [60] as a probe (Figure 1). As expected, all chromosome ends were labelled. No strong signals were observed in nonterminal positions, indicating that, like in the human genome, het-ITS are not present in the horse genome. Only some faint intrachromosomal signals were detected (arrows in Figure 1). Based on our previous work [24,29], we conclude that these faint signals correspond to short-ITSs. However, as demonstrated previously, the FISH technique is not sensitive enough to efficiently detect short sequences. A human spread hybridized with the same telomeric probe, showing a similar pattern of faint interstitial signals corresponding to short ITSs, is shown in a previous publication [13].

To obtain a comprehensive catalogue of horse short-ITSs we carried out a BLAST search in the reference genome sequence of *Equus caballus* (EquCab3.0) [61] that was obtained by the assembly of the genomic sequence of the thoroughbred mare Twilight, the same individual used to obtain the previous horse genome assembly EquCab2.0 [52]. We used the parameters described above for searching human ITSs, i.e., the presence of at least four TTAGGG units with less than one mismatch per unit. Using this procedure, we identified 140 loci. As described in the following paragraph, two additional loci were found in the trace database, bringing the total number of ITSs to 142. In Supplementary Table S3 these loci are listed together with their length and number of mismatches. It is possible that additional ITS loci, represented by homozygous empty alleles in Twilight, may be present in the horse population.

### 2.3. Search of ITS-Less Alleles in Twilight

To identify horse ITS-less alleles, we carried out a search of heterozygous loci in the genome of Twilight using the two strategies summarized in Supplementary Figure S1. 

Since only one allele per locus is included in the reference genome, according to the first strategy, we used the ITS loci as query to BLAST search possible corresponding empty alleles in the NCBI Trace Database (https://blast.ncbi.nlm.nih.gov/Blast.cgi?PAGE=Nucleotides&PROGRAM=blastn&BLAST_SPEC=TraceArchive&BLAST_PROGRAMS=megaBlast&PAGE_TYPE=BlastSearch) [62], which includes unassembled DNA sequences from Twilight. Using this method, we found three ITS-less alleles corresponding to chr15:23487997, chr19:32741 and chr27:21217687. Since this method would not allow us to identify heterozygous ITS loci whose empty allele was included in the assembled reference genome, according to the second strategy, we BLAST searched loci containing telomeric repeats in the horse Trace Database. Using this second procedure, we found two additional ITS loci that were not found in the assembled reference genome (chr2:13178780 and chr9:1570127). These loci are heterozygous in Twilight. Altogether, we identified 142 ITS loci, five of which are heterozygous in Twilight. In Supplementary Table S3, horse ITS loci are listed. 

**Figure 2:**
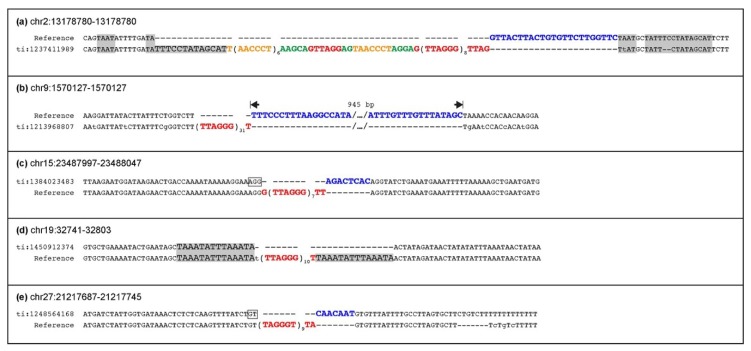
ITS loci heterozygous in Twilight. For each locus, sequence alignment of the empty allele (top) with the ITS-containing allele (bottom) is shown. The sequence indicated as Reference corresponds to the allele found in the horse reference genome EquCab3.0, the alternative allele is indicated by the NCBI Trace ID number. Telomeric repeats in TTAGGG and CCCTAA orientation are indicated in red and orange, respectively. Flanking sequence modifications that occurred together with telomeric repeat insertions are indicated using different colors: nucleotides deleted from the flanking sequence are in blue; random nucleotides sequence insertions are in green; duplications of the insertion site are shaded in grey. Nucleotides at break sites in register with the inserted telomeric repeats are boxed.

In Figure 2, the sequences of the ITS-containing and of the ITS-less alleles of the five heterozygous loci are reported. At four of these loci, the insertion of telomeric repeats was accompanied by the deletion of a sequence flanking one side of the break (Figure 2a–c,e). At two loci (Figure 2a,d), the direct repetition of a sequence flanking the break was generated. In addition, at the third and fourth of these loci (Figure 2c,d), we detected two and three nucleotides in register with the inserted telomeric repeat, respectively. These nucleotides correspond to microhomology to the telomeric hexamer at the 3’ end of the break site.

We can reasonably predict that the five loci that are heterozygous in Twilight are polymorphic and that a large number of additional polymorphic loci are present in the horse population.

### 2.4. Polymorphism of Horse ITSs

Three of the heterozygous ITS loci identified in Twilight were analyzed by PCR in 114 horses from five breeds (Norwegian Fjord, Icelandic Pony, Quarter Horse, Andalusian and Lipizzaner), from 18 Show Jumping horses and from 20 Przewalski’s horses. Genomic DNA was amplified using the primers listed in Supplementary Table S4. Table 2 shows that the frequency of the empty allele is variable among the different populations, ranging from 0.47 to 1.00. Empty alleles tend to be well-represented in all populations analyzed, being either more or equally frequent compared to ITS alleles. At the locus on chromosome 19, the ITS-containing allele was found, at a low frequency, only in Show Jumpers and Quarter horses, while it was absent in the other populations. While the other two ITSs seem to be absent in Przewalski’s horses, the locus on chromosome 15 is polymorphic also in this species.

In previous work, we observed that several human ITS loci are characterized by variable number of tandem repeats (VNTR) polymorphism [63]. To test whether this type of variability is also present at horse ITSs, we analyzed 11 ITS loci in the 18 Show Jumping horses (Table 3). This analysis includes the three loci already characterized for insertion polymorphism (Table 2). At eight loci, more than one VNTR allele was found, with the number of alleles ranging from two to six. At two loci (chr2:13178780, chr15:23487997), both insertion and VNTR polymorphism was detected.

### 2.5. Comparison of ITS-Containing and ITS-Less Sequences: Mechanisms of Telomeric Repeat Insertion

In previous work we demonstrated that, in primates and rodents, interstitial telomeric repeats were inserted in one step in the course of evolution. A comparative analysis of the sequences flanking the telomeric repeats with the sequence of orthologous empty loci in evolutionarily related species had allowed us to demonstrate that the insertion sites often underwent the typical modifications occurring during nonhomologous end-joining [25,27]. This analysis also strongly suggested that telomerase was involved in this pathway [25,27]. 

To identify ITS-less ancestral loci orthologous to horse ITSs, we used 1 kb sequences containing each horse ITS as query for a BLAST search against the draft genomic sequences of donkey (*Equus asinus*) [64,65] and white rhinoceros (*Ceratotherium simum simum*) that are available at the NCBI genome database (https://www.ncbi.nlm.nih.gov/assembly/GCF_001305755.1; https://www.ncbi.nlm.nih.gov/assembly/GCA_003033725.1; https://www.ncbi.nlm.nih.gov/assembly/GCF_000283155.1) [66,67,68]. For 46 of the 142 horse ITS loci, the telomeric repeat was conserved in the three species (Table 4). For 66 ITS loci, we found orthologous empty loci in donkey and/or rhinoceros. For 30 horse ITSs, the orthologous loci in the other two species were not detectable due to gaps in the genome assembly or to gross sequence rearrangements. Four of the five loci for which Twilight is heterozygous (marked with an asterisk in Table 4) are empty in the other two species, confirming that they were inserted recently in the horse lineage. At the fifth locus, a telomeric repeat stretch is present in the orthologous donkey locus, suggesting that lineage sorting may have occurred in the common ancestor of the horse and donkey lineages.

**Figure 3:**
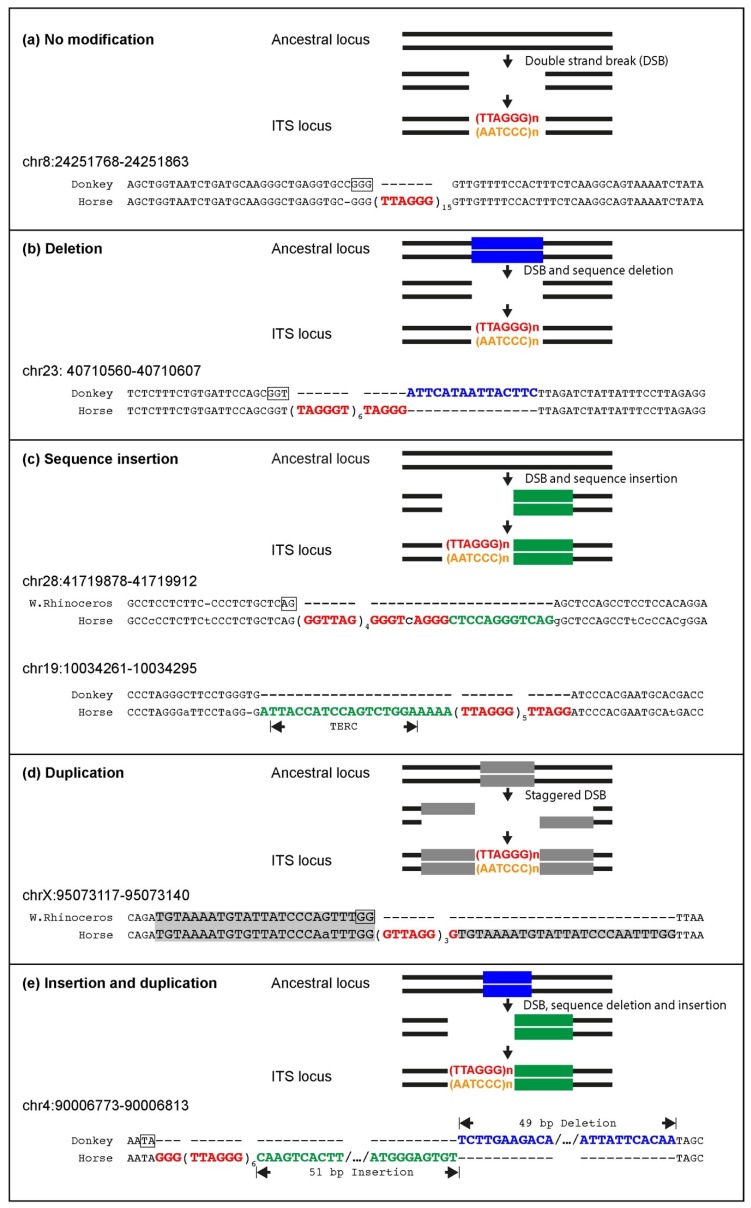
Examples of data used to describe the insertion mechanism of telomeric repeats. For each locus, the alignment of the empty ancestral sequence from donkey or white rhinoceros with the ITS sequence in the horse reference genome assembly is shown. A sketch of the mechanism responsible for telomeric repeat insertion is shown on top of each sequence alignment. Telomeric repeats in TTAGGG and CCCTAA orientation are indicated in red and orange, respectively. At empty loci, nucleotides in register with the inserted telomeric repeats are boxed. (**a**) Interstitial telomeric repeat insertion occurred without modification of the sequences flanking the double-strand break. The orthologous locus from donkey is empty at the insertion site. The double-strand break exposed a GGG trinucleotide in register with the inserted telomeric repeats. (**b**) Interstitial telomeric repeat insertion accompanied by the deletion of nucleotides from the flanking sequence (blue nucleotides, blue strip in sketch). The double-strand break exposed a GGT trinucleotide in register with the inserted telomeric repeats. (**c**) Interstitial telomeric repeat insertion accompanied by the addition of a nucleotide sequence (green nucleotides, green strip in sketch). Telomeric repeat insertion was accompanied by the addition of a random nucleotide sequence, and the double-strand break exposed an AG dinucleotide in register with the inserted telomeric repeats. The ITS was inserted together with 17 bp homologous to a region of horse TERC 91 nucleotides away from the telomeric repeat template. (**d**) Interstitial telomeric repeat insertion at a staggered double-strand break followed by flanking sequence duplication (nucleotides shaded in grey, grey strip in sketch). The double-strand break exposed a GG dinucleotide in register with the inserted telomeric repeats. (**e**) Interstitial telomeric repeat insertion accompanied by a complex modification of the insertion site involving the simultaneous deletion of nucleotides and addition of a random sequence. A TA dinucleotide in register with the inserted telomeric repeats was exposed by the double-strand break.

**Figure 4:**
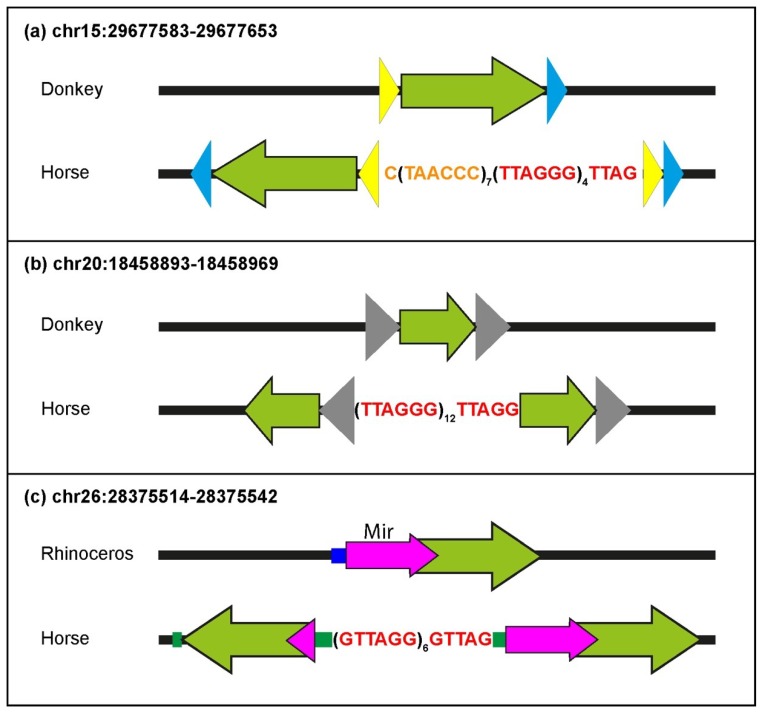
ITS insertions accompanied by complex rearrangements. Telomeric repeat insertions were accompanied by complex modifications of the break site involving addition of random nucleotide sequences, deletion of nucleotides from the insertion site, inversions or generation of inverted duplications. (**a**) Telomeric repeat insertion was accompanied by inversion of a single copy sequence (light green arrow) and generation of inverted duplications (yellow and light blue arrowheads) of sequences present at the empty locus. The rearrangement also caused the formation of telomeric stretches with head-to-head orientation. (**b**) The ancestral ITS-less locus from donkey contains a single copy sequence (light green arrow) flanked by short direct repeats (grey arrowheads). Telomeric repeat insertion at the horse locus was accompanied by duplication and inversion of the upstream direct repeat and of the single copy sequence. (**c**) The empty locus in rhinoceros contains a Mammalian-wide interspersed repeat (Mir, purple arrow) and a single copy sequence (light green arrow). Telomeric repeat insertion was accompanied by deletion of a small sequence from the insertion site (blue strip), duplication and inversion of the single copy sequence and of a portion of the Mir and insertion of small random nucleotide sequences (green strips).

In Figure 3, examples of sequence comparisons between ITS-containing and their corresponding empty loci are shown. At the locus shown in Figure 3a, the insertion of telomeric repeats occurred without modification of the target sequence, whereas in Figure 3b the deletion of a short sequence from the insertion site accompanied ITS insertion. In Figure 3c, the sequence of two loci is shown. At chr28:41719878, the ITS was introduced together with an apparently random sequence, whereas at chr19:10034261, 17 nucleotides retrotranscribed from the horse telomerase RNA were inserted. In Figure 3d, a direct duplication of the target sequence is shown. In the ITS shown in Figure 3e, a deletion and a random sequence insertion occurred together with the telomeric repeat insertion. The generation of three ITS loci was accompanied by complex rearrangements that are sketched in Figure 4. The first rearrangement (Figure 4a) involved the inversion of a 286 bp sequence and the inverted duplication of two short sequences (38 and 53 bp). This rearrangement created two head-to-head stretches of the telomeric sequence. Inversions and duplications of sequences flanking the site of telomeric repeat insertion generated the ITS shown in Figure 4b. The generation of the ITS in Figure 4c involved insertions, a deletion and an inverted duplication.

The frequency of the different types of modifications at ITS insertion sites is reported in Table 5. In about 17% of the loci, the telomeric repeat was inserted without any sequence modification at the break site. The deletion of short sequences from the insertion site was the most frequent modification (30%), while complex rearrangements occurred at the insertion site in about 29% of the events.

A relevant observation deriving from this comparative analysis was the nonrandom presence of nucleotides in register with the inserted telomeric sequence in the ancestral ITS-less loci, In the examples shown in Figure 2; Figure 3, such nucleotides are highlighted. For this analysis, we could only utilize 40 loci where the ancestral sequence flanking the ITS was not modified during telomeric repeat insertion. This sequence arrangement was observed at 31 out of the 40 informative loci (Table 6). About 78% of the ITSs were inserted at sites where 1-6 nucleotides in register with the telomeric repeats were exposed at the 3’ end of the double-stranded DNA break. This value is much higher than expected by randomness (≤25%). Even more striking is the difference between observed and expected values when we consider loci with two or more nucleotides in register (Table 6).

### 2.6. Conservation and Genome Distribution of ITSs

To study the conservation between horse and human ITSs, only the 46 horse ITSs that are conserved in donkey and rhinoceros were analyzed (Table 4), while species- or genus-specific ITSs were not considered informative for this analysis. The analysis was carried out using BLAT to compare the horse and the human orthologous loci. For two of the horse ITSs, orthologous ITS loci were present in the list of the 229 human ITSs containing at least four telomeric repeats and less than one mismatch per unit (chr10:81331655-81331689 and chr26:32420315-32420368). For four additional horse ITSs, orthologous ITS loci were found in the human reference genome; however, since their sequence was degenerate, they were not comprised in our list (chr1:91725039-91725121, chr2:19293258-19293287, chr3:68906105-68906142 and chr15:77022476-77022502). 

None of the human ITSs were contained within exons, while 31% of them were contained in introns of coding NCBI annotated genes. Given the incomplete annotation of the horse genome, this analysis could not be performed in the horse.

In Supplementary Tables S5 and S6, the distribution of ITSs on all human and horse chromosomes, respectively, is shown. In the tables, the average number of ITSs per Mb on each chromosome is also shown.

## 3. Discussion

We previously classified interstitial telomeres according to their cytogenetic position and sequence organization as heterochromatic, short, fusion and subtelomeric [13].

In previous studies, large blocks of telomeric-like repeats, corresponding to heterochromatic ITSs, could be detected by FISH in several metazoan and plant species [14,15,16,17,18,19,20]. The application of the FISH technique revealed that this type of ITS is not present in the human genome, while allowing us to detect only a limited number of short-ITSs [24]. In the present work, the same kind of analysis applied to horse metaphase spreads revealed that the general organization of interstitial telomeres in horses is similar to the one described in humans. As for the human situation, the horse short-ITSs were displayed as weak signals or remained largely undetected, due to the limited sensitivity of the FISH technique.

To compile a comprehensive list of short-ITSs in the human and horse genomes and to study their sequence organization, we analyzed the genome assemblies of the two species. It is worth mentioning that the number of ITSs that can be detected with this approach depends on the software, parameters used and coverage of the genome assembly. For instance, by BLAT search against the human genome version NCBI34/hg16, we previously found 83 ITSs composed by at least four repeats with less than one mismatch per repetition [25]. In a successive work, in which we discovered that telomeric repeat factors 1 and 2 (TRF1 and TRF2), which are the two main telomere binding proteins involved in telomere structure and function, bind to a subset of interstitial telomeric repeats, a less stringent search of ITSs was carried out using the automatic RepeatMasker annotation [69]. Following this search, we found 714 loci which included highly degenerate telomeric-like repeats. In that study we used pre-masked genome data from the software RepeatMasker, which tends to split long or degenerate repeat arrays into several shorter hits. In the present work, we used BLAST to carry out a search in the hg19/GRCh37 version of the human genome assembly, manually corrected overlapping hits and discarded degenerate repeat arrays. With this strategy, we identified 229 human ITSs containing at least four TTAGGG repeats with less than one mismatch per repeat. Using the same approach, we identified 142 short-ITSs in the horse reference genome. We have chosen to consider only stretches of at least four TTAGGG repeats with less than one mismatch per unit to avoid detection of short sequences possibly occurring in the genome by chance. A number of shorter and/or more degenerate ITSs are not included in our list. The choice of these parameters was arbitrary. In previous work, we demonstrated that, following their insertion, telomeric repeats undergo mutation during evolution; therefore, “young ITSs” are characterized by greater sequence conservation compared to “old ITSs” [25]. Since we were interested in finding insertion polymorphism and in describing insertion mechanisms, we concentrated our analysis on well-conserved and not too short “young” ITSs. It is noteworthy that one ITS in the human genome, at chromosome 2q13, was derived by fusion between ancestral acrocentric chromosomes [22], while we could not find any evidence of such ITS type in the horse.

A comparative analysis between human and horse ITSs showed that six horse ITSs have been inserted in the genome of a common ancestor of Primates and Perissodactyla, more than 90 million years ago [70]. It would be interesting to test whether the conservation of the telomeric repeat during such an extended evolutionary time may be related to any function. None of the human ITSs were inserted into exons of coding genes. This result is not surprising because such mutation would have inserted stop codons in both orientations.

The distribution of human and horse ITSs along chromosomes does not seem to be related to their size but is probably the result of random insertions. The fraction of human ITSs localized within introns (31%) is compatible with their random insertion in the genome since the fraction of human genome occupied by introns has been estimated to be between 26% and 38% [71]. It will be interesting to test whether the presence of telomeric repeats within introns may affect splicing.

In previous studies, we demonstrated that short-ITSs were introduced in one step at a given time during the evolution of primate and rodent lineages [25,27]. Therefore, short-ITSs can be considered insertion sequences. 

It is well-known that insertion sequences that were introduced recently during evolution can display insertion polymorphism [36,37,38,39,40,41,42,49,50]. That is to say that the insertion-containing allele is not yet fixed, and the empty ancestral allele is also present in the population. Sequences showing insertion polymorphism have been used as markers for population genetic studies in many species including humans [37,42,47,49] and, in some cases, they have been associated to gene expression regulation [43,49]. To our knowledge, insertion polymorphism at short-ITS loci has not been described so far. Given the short length of these repeated arrays, their variation cannot be detected by FISH but only by sequence analysis. Indeed, only a fraction of short ITSs can be detected by FISH as faint signals whose frequency is related to the number of repeats at each locus (Figure 1) [24,29]. On the contrary, variation of het-ITSs was described before through FISH experiments in plants [72] and in PALA (N-(phosphonacetyl)-L-aspartate)-resistant CHO cells containing amplifications of the CAD (carbamyl-P-synthetase, aspartate transcarbamilase, dihydro-orotase) gene [60].

Are short-ITSs inserted at random sites or within specific genomic regions? To answer this question, we analyzed the GC content of the regions surrounding human and horse short-ITSs. The analysis was carried out within windows of different length: 100 bp, 1 kb and 5 kb on each side of the telomeric repeat. The values varied greatly among different loci, ranging between 12% and 75%, and the average values corresponded to 41.6% and 41.9% in horse and human, respectively (data not shown). We could conclude that there is no preferential choice for ITS insertion based on GC content. In previous studies we showed that, in primates and rodents, ITS colocalize with fragile sites [26,28,29,35]. Although we do not know which genomic or epigenetic features may be related to the fragility of these sites, this correlation strongly supported the model of ITS insertion at DNA double-strand break sites.

In this work, we searched for the presence of ITS insertion polymorphism in the human population. Surprisingly, despite the large number of individuals analyzed, no ITS-less alleles were found, suggesting that this kind of polymorphism is not present, although we cannot exclude that very rare ITS-less alleles at some loci may exist.

We have previously shown that, in the horse, insertion polymorphism is particularly frequent for numts and ERE1 transposable elements [49,50]. We wondered whether loci polymorphic for ITS insertion could be detected as well. Indeed, as opposed to what we observed in humans, we found five ITS loci heterozygous for the presence of telomeric repeats in the genome of a single horse individual: the mare Twilight, who donated her DNA for the reference genome assembly. A PCR analysis of three loci heterozygous in Twilight in six horse breeds and in Przewalski’s horses confirmed that they are polymorphic. The ITS at chr15:23487997 is polymorphic both in *Equus caballus* and in *Equus przewalskii*, suggesting that the insertion of the telomeric repeat stretch pre-dates the separation of the domestic and Przewalski’s horse lineages. Therefore, this ITS was inserted in the genome of the common ancestor of the two horse lineages more than 0.5 million years ago [51]. For the ITSs at chr2:13178780 and at chr19:32741, all analyzed individuals from Przewalski’s horse were homozygous for the ITS-less allele, suggesting that the insertion of telomeric repeats at these loci may have occurred in the domestic horse lineage very recently, after its separation from the Przewalski’s horse lineage. Alternatively, since the population of modern Przewalski’s horses derives from a few individuals [73], the absence of the ITS may be due to genetic drift.

Our results underline a striking difference between the human and horse genomes in terms of ITS insertion polymorphism. In humans, such polymorphism is either absent or very rare, while our data strongly suggest that it is extremely frequent in the horse. As mentioned above, in the horse, insertion polymorphism is also very frequent for ERE1 retrotransposons and numts. All together, these findings provide further evidence to the notion that the horse genome is in a stage of rapid evolution. In line with this hypothesis is our discovery of an evolutionary new centromere, totally devoid of satellite tandem repeats, on horse chromosome 11 [52,58]. Therefore, different molecular mechanisms, such as transposition, DNA double-strand break repair and centromere repositioning contribute to the great plasticity of the horse genome in the current evolutionary stage.

We previously described polymorphism of human ITS loci due to variable number of tandem repeats [63]. VNTR polymorphism is also present at horse ITSs, indicating that this peculiar type of microsatellite can be unstable and that, similarly to microsatellites with shorter units, they may be useful polymorphic markers for linkage analysis and parentage testing.

### Mechanisms of ITS Insertion in the Horse Genome

Several ITSs were inserted in the horse genome within target sequences that are well-conserved in the orthologous position of the donkey or rhinoceros genome. Therefore, similarly to primates and rodents, ITS insertions have also occurred in one step in equids [25,27]. In the present work, the comparison between ITSs and ITS-less ancestral orthologous loci allowed us to demonstrate that the insertion sites underwent modifications that are typical of the nonhomologous end-joining pathway, supporting our previous hypothesis that they are generated in the course of evolution during the repair of DNA double-strand breaks [25,27]. Deletions of short sequences are the most frequent modifications occurring at the break site during the insertion of ITSs, but random sequence addition also occurred. At a few horse ITS loci, direct duplications of target sequences occurred that are likely resulting from the repair of staggered double-strand DNA breaks. Deletions of sequences flanking the break site were indeed the most frequent modifications observed at junctions produced by the repair of double-strand breaks (DSB) induced in experimental systems, while additions and duplications were also observed [74,75,76]. During DSB repair, sequence modifications of the broken ends seem to be often necessary to provide the correct substrate for the final ligation reaction and are operated by specific enzymes such as the Mre11, Exo1 and Artemis nucleases, polynucleotide kinases and template-independent DNA polymerases [77]. Interestingly, the insertion of telomeric repeats in the horse genome was frequently accompanied by complex modifications of the target sequence involving combinations of deletions, additions, inversions and duplications. Such complex rearrangements were not observed in our previous analysis of rodents and primates, further confirming the great plasticity of the horse genome. In one ITS (chr19:10034261, Figure 3c), the telomeric repeat stretch was inserted together with a sequence retrotranscribed from a region of the telomerase RNA component (TERC) far away from the telomeric template. Our previous observation of 14 mouse ITS loci with a similar sequence arrangement, called TERC-ITS [25], provided a strong indication that the telomerase enzyme may be involved in the generation of interstitial telomeres. Having also found a TERC-ITS in the horse genome corroborates this interpretation. Further evidence supporting the involvement of telomerase in ITS insertion is the observation that, in a highly significant number of ITS loci, nucleotides in register with the telomeric repeat sequence were exposed at the break site that occurred in the ancestral sequence. In this scenario, ITS insertion represents one of the noncanonical and controversial roles of telomerase that have been recently proposed [78]. An alternative mechanism that may account for the generation of some ITSs relies on the introduction of retrotranscribed telomeric RNA into DNA double-strand break sites. These two proposed pathways may be activated in different conditions.

To test whether ITSs can be introduced at DNA double-strand break sites in somatic cells in culture, we previously set up an experimental system based on the induction of site-specific breaks by the I-SceI endonuclease [75]. We analyzed about 350,000 junctions generated by the repair of these breaks but never observed the insertion of a telomeric repeat stretch, suggesting that, in this system, this event is very rare or does not occur at all. In a successive work, Onozawa and colleagues [76] transfected total RNA into cultured human cancer cells in which double-strand breaks were induced at I-SceI sites and showed that sequences retrotranscribed from the RNA could be introduced at the break site. At four of these insertions they found telomeric repeats and suggested that these sequences may have been retrotranscribed from telomerase RNA. We can now suggest that the telomeric repeats observed in this experimental system may have been retrotranscribed from TERRA, the family of RNA molecules transcribed from telomeres. It is tempting to postulate that also in vivo some ITSs may have been generated in the germ-line by a DNA repair pathway involving the insertion of DNA fragments retrotranscribed from TERRA molecules.

## 4. Materials and Methods

### 4.1. Search of ITS in the Human and Horse Genome Sequence

To identify human ITSs, the sequence (TTAGGG)_4_ was used as query for a BLAST search against the genome reference sequence hg19/GRCh37.p13 (https://blast.ncbi.nlm.nih.gov/Blast.cgi?PAGE_TYPE=BlastSearch&BLAST_SPEC=OGP__9606__9558&LINK_LOC=blasthome) [79]. The search was performed using the “blastn” algorithm and the standard setup. The automatic adjustment of search parameters for short sequences was disabled. The BLAST search produced 3689 hits. Hits mapping on patches and unplaced sequences were removed, leaving 2970 hits. Further manipulations of the hit list were carried out using tools available on the Galaxy platform (https://usegalaxy.org/) [80,81]. To reconstruct the full sequence of ITS loci, hits with overlapping coordinates were merged into single loci using the “Merge the overlapping intervals of a dataset 1.0.0” tool.

Manual analysis of hits showed that BLAST splits long or degenerate ITSs into several shorter loci, causing an overestimation of the number of ITSs. To overcome this problem, we merged these hits into single loci using the function “Cluster the intervals of a dataset 1.0.0” followed by “Merge the overlapping intervals of a dataset 1.0.0”, leaving 555 hits. We manually checked each locus of the list to remove false positives (telomeres or GC-rich stretches), leaving 458 loci. Finally, we selected sequences composed by at least four telomeric repeats and no more than 1.0 mismatch per unit, leaving 229 short human ITSs.

To identify horse ITSs, we applied the same search protocol to the horse reference genome sequence (NCBI horse genome sequence EquCab3.0). The BLAST search produced 10,328 hits. Hits positioned on unplaced chromosomes were removed, leaving a total of 7651. Removal of overlapping hits, merging of split hits into single loci and manual check left 306 ITSs. Finally, we selected sequences composed by at least four telomeric repeats and no more than 1.0 mismatch per unit, leaving 140 short ITSs.

### 4.2. In Silico Search of ITS Insertion Polymorphism in the Human Population

In order to identify empty alleles in the human population, we checked the 229 human ITSs in the 2504 genome sequences that were produced for the 1000 Genome Project. Empty alleles were searched using the UCSC Genome Browser and track “1000 Genomes Phase 3 Integrated Variant Calls: SNVs, Indels, SVs” (https://genome.ucsc.edu/cgi-bin/hgTrackUi?hgsid=720970681_qqmhEowWab8OoZ9mluPovptBdLXW&c=chrX&g=tgpPhase3) [82].

### 4.3. PCR Amplification of Four Human-Specific ITS Loci in Individuals from Different Countries

Human genomic DNA samples (50–100 ng) were previously used in Semino and colleagues [83]. PCR reactions were performed in a 25 μL final volume with 20 pmol of each primer, 0.2 mM dNTP, 1X Green Buffer (Promega Italia, Milano, Italy), 0.5 units of GoTaq DNA polymerase (Promega Italia, Milano, Italy) and water. After a denaturation step at 95 °C for 2 min, the following amplification cycle was performed 35 times: 95 °C for 40 s, annealing at the appropriate temperature for 40 s, 72 °C for 30 s. Final extension was carried out at 72 °C for 5 min. PCR products were checked by electrophoresis in 1–2% agarose gel. PCR primers are listed in Supplementary Table S2.

### 4.4. Identification of Empty ITS Loci in the Horse Reference Genome

To identify loci that are heterozygous in the reference genome, we screened the Horse Whole Genome Shotgun sequences in the NCBI Trace Database, which includes unassembled DNA sequences from Twilight. We downloaded 2 kb sequences containing the horse ITS loci from UCSC Genome Browser (https://genome.ucsc.edu/cgi-bin/hgGateway) [84], and then we manually removed the telomeric repeats. The “ITS-less” sequences were used as queries for a BLAST search against the Horse Whole Genome Shotgun sequence Trace Database (Database: Equus caballus—WGS, Title: equus_caballus, Molecule Type: Genomic, Update date: 2010/01/15) (https://blast.ncbi.nlm.nih.gov/Blast.cgi?PAGE=Nucleotides&PROGRAM=blastn&BLAST_SPEC=TraceArchive&BLAST_PROGRAMS=megaBlast&PAGE_TYPE=BlastSearch) [62] using the “blastn” algorithm and the standard setup. The automatic adjustment of search parameters for short sequences was disabled.

To identify heterozygous ITS loci whose empty allele was included in the assembled reference genome, we used the sequence (TTAGGG)_4_ as query to perform a BLAST search against the Horse Whole Genome Shotgun sequence Trace Database (Database: Equus caballus—WGS, Title: equus_caballus, Molecule Type: Genomic, Update date: 2010/01/15) (https://blast.ncbi.nlm.nih.gov/Blast.cgi?PAGE=Nucleotides&PROGRAM=blastn&BLAST_SPEC=TraceArchive&BLAST_PROGRAMS=megaBlast&PAGE_TYPE=BlastSearch) [62]. Trace sequences were downloaded and used as query for a BLAST search against the horse genome reference sequence (NCBI horse genome sequence EquCab3.0) using the “blastn” algorithm.

### 4.5. PCR Amplification in Horse Populations

Genomic DNAs from 18 Show Jumping horses were prepared from peripheral blood samples of individuals that, according to their pedigree chart, do not share common ancestors up to the third generation. The genomic DNA samples were previously used in another study [49], thus sampling was not required for this work.

DNA samples from Quarter horses, Andalusian horses, Norwegian Fjords, Icelandic ponies and *E. przewalskii* were provided by Professor Cecilia Penedo (UC Davis, Davis, CA, USA). Professor Ernest Bailey (Gluck Equine Research Center, University of Kentucky, Lexington, KY, USA) provided DNA samples from Andalusian horses and Icelandic ponies. Lipizzaner DNA samples were described in [85].

PCR reactions were carried out as described above. For each locus, primer pairs were designed on the sequences flanking the telomeric repeat. PCR primers are listed in Supplementary Table S4.

### 4.6. Identification of ITS-less Loci in Equus Asinus and Ceratotherium Simum Simum

For each horse locus, we downloaded a 1 kb sequence containing the ITS, 500 bp from the 5’ flanking region and 500 bp from the 3’ flanking region. The sequence was used as query for a BLAST search against the donkey genome sequences published by Huang and collaborators (https://www.ncbi.nlm.nih.gov/assembly/GCF_001305755.1) [64,66] and by Renaud and collaborators (https://www.ncbi.nlm.nih.gov/assembly/GCA_003033725.1) [65,67]. The same strategy was used to identify empty ancestral loci in the genomic sequence of white rhinoceros (cerSim1/CerSimSim1.0, assembly version May 2012; https://www.ncbi.nlm.nih.gov/assembly/GCF_000283155.1/) [68].

Sequences were compared using the Multalin software (http://multalin.toulouse.inra.fr/multalin/) [86,87].

### 4.7. Cell Culture and Fluorescence In Situ Hybridization

Horse primary fibroblasts were previously isolated and established from skin samples of slaughtered animals under sterile conditions [53,88].

Cells were cultured in high-glucose Dulbecco’s modified Eagle’s medium (EuroClone, Pero, Italy) supplemented with 20% fetal bovine serum (EuroClone, Pero, Italy), 2 mM glutamine (Sigma-Aldrich, St. Louis, MO, USA), 2% nonessential amino acids (EuroClone, Pero, Italy) and 1X penicillin/streptomycin (Sigma-Aldrich) at 37 °C in a humidified atmosphere of 5% CO_2_. Metaphase spreads were prepared as previously described [89].

The telomeric probe is a mixture of 1-20 kb long synthetic (TTAGGG)n fragments that was previously prepared in our laboratory [24,60] and labelled by nick translation with Cy3-dUTP (Enzo Life Sciences, Farmingdale, NY, USA). Hybridization to metaphase spreads and post-hybridization washes were carried out in low-stringency conditions as previously described [89]. Chromosomes were counterstained with 0.2 μg/mL DAPI and mounted with DAKO mounting medium. Digital images of fluorescence signals were acquired with a fluorescence microscope (Zeiss Axioplan) equipped with a cooled CCD camera (Photometrics). Pseudocoloring and merging of images were performed using the IPLab 3.5.5 Imaging Software (Scanalytics inc., Fairfax, VA, USA). To acquire images of metaphase spreads, a 63× objective was used. For the images shown in Figure 1A-J, 2× enlargements of portions of the spreads were obtained using Adobe Photoshop CS6.

## 5. Conclusions

The human and horse genomes showed a striking difference in terms of ITS insertion polymorphism: in humans, such polymorphism is either absent or very rare, while it is extremely frequent in the horse. These observations support the hypothesis that the horse genome is in a stage of rapid evolution.

Through sequence comparison between horse ITSs and their corresponding empty loci we analyzed the molecular mechanisms of their insertion during evolution. The results allowed us to describe several types of rearrangements deriving from the processing of DNA ends that occurred together with telomeric repeat insertion, providing compelling evidence to the conclusion that short-ITSs are generated by a DNA double-strand break repair pathway.

## Figures and Tables

**Table 1:** Human-specific interstitial telomeric sequence (ITS) loci analyzed for insertion polymorphism. Coordinates of the loci, country of origin and number of individuals analyzed for each locus are indicated. NA, not assayed.

	Locus, Number of Individuals							
	chr3:159412014-159412054		chr11:129573442-129573473		chr15:42243201-42243234		chr16:75368098-75368138	
Macro Area (Country/Population)	No. of Individuals	No. of Empty Loci	No. of Individuals	No. of Empty Loci	No. of Individuals	No. of Empty Loci	No. of Individuals	No. of Empty Loci
West Africa (Senegal/Wolof)	17	0	25	0	24	0	22	0
West Africa (Senegal/Mandenka)	22	0	26	0	28	0	28	0
East Africa (Kenya)	10	0	9	0	10	0	11	0
East Africa (Ethiopia)	11	0	13	0	12	0	13	0
Near East (Lebanon)	31	0	NA	NA	31	0	30	0
Asia (Turkey)	17	0	NA	NA	20	0	14	0
Asia (China/Han)	6	0	11	0	7	0	7	0
Europe (Croatia)	11	0	NA	NA	11	0	10	0
Europe (Hungary)	6	0	NA	NA	9	0	7	0
Europe (Italy)	19	0	12	0	26	0	23	0
Europe (Poland)	15	0	10	0	13	0	11	0
Europe (Ukraine)	10	0	10	0	8	0	10	0
Europe (Others)	12	0	11	0	10	0	10	0
**Total**	187	0	127	0	209	0	196	0

**Table 2:** Insertion polymorphism of ITS loci in seven horse populations.

		Frequency of the Empty Allele		
Population	Number of Individuals	chr2:13178780-13178780	chr15:23487997-23488047	chr19:32741-32803
Show Jumping horse	18	0.53	0.53	0.89
Quarter horse	20	0.47	0.62	0.87
Lipizzaner	23	0.83	0.96	1.00
Norwegian Fjord	20	0.85	0.63	1.00
Andalusian	25	0.66	0.56	1.00
Icelandic pony	26	1.00	0.85	1.00
Przewalski’s horse	20	1.00	0.75	1.00

**Table 3:** Variable number of tandem repeats at 11 ITS loci in 18 horses.

ITS Locus	No. of Telomeric Repeats	Frequency
**chr20:18458893**		
Allele 1	11	0.03
Allele 2	12	0.14
Allele 3	13	0.61
Allele 4	14	0.11
Allele 5	15	0.08
Allele 6	16	0.03
**chr23:44283331**		
Allele 1	5	0.03
Allele 2	7	0.31
Allele 3	8	0.25
Allele 4	9	0.36
Allele 5	10	0.06
**chr19:10034261**		
Allele 1	6	0.72
Allele 2	7	0.11
Allele 3	8	0.17
**chr27:29189054**		
Allele 1	5	0.06
Allele 2	7	0.78
Allele 3	8	0.17
**chr25:15560895**		
Allele 1	9	0.72
Allele 2	10	0.28
**chr29:23755261**		
Allele 1	7	0.11
Allele 2	8	0.89
**chr5:90360200**		
Allele 1	6	1.00
**chr8:24251768**		
Allele 1	16	1.00
**chr2:13178780**		
Allele 1	0	0.53
Allele 2	15	0.11
Allele 3	16	0.31
Allele 4	17	0.06
**chr15:23487997**		
Allele 1	0	0.53
Allele 2	6	0.17
Allele 3	7	0.31
**chr19:32741**		
Allele 1	0	0.89
Allele 2	10	0.11

**Table 4:** Horse ITS loci informative for the mechanisms of telomeric repeat insertion. Loci are ranked by coordinate. Legend: ITS, telomeric repeats are present at the insertion site; Empty, the orthologous locus is empty at the insertion site; NF, orthologous locus not found.

	Loci Orthologous to Horse ITS	
Horse ITS Locus	Donkey	White Rhinoceros
chr1:27309501-27309532	ITS	Empty
chr1:90843617-90843649	Empty	Empty
chr1:98488870-98488928	ITS	Empty
chr1:156995264-156995361	ITS	Empty
chr2:661976-662028	ITS	Empty
chr2:13178780-13178780 *	Empty	Empty
chr2:30174747-30174793	ITS	Empty
chr3:6047114-6047139	ITS	Empty
chr3:45427234-45427258	ITS	Empty
chr3:82188585-82188664	ITS	Empty
chr3:92686382-92686405	ITS	Empty
chr3:106340791-106340815	ITS	Empty
chr4:65502437-65502468	ITS	Empty
chr4:68540207-68540247	ITS	Empty
chr4:81320764-81320806	ITS	Empty
chr4:90006773-90006813	Empty	NF
chr5:36007702-36007752	Empty	NF
chr5:88367091-88367115	ITS	Empty
chr6:8289417-8289447	ITS	Empty
chr6:38922364-38922401	ITS	Empty
chr7:29297817-29297852	ITS	Empty
chr7:51713092-51713124	ITS	Empty
chr7:100764221-100764245	ITS	Empty
chr8:24251768-24251863	Empty	Empty
chr8:64629752-64629778	ITS	Empty
chr8:94527922-94527948	ITS	Empty
chr9:1570127-1570127 *	Empty	Empty
chr9:74200299-74200328	ITS	Empty
chr9:80878399-80878422	ITS	Empty
chr10:7134943-7135113	ITS	Empty
chr10:22622806-22622833	ITS	Empty
chr11:42736447-42736474	ITS	Empty
chr11:44810227-44810253	ITS	Empty
chr12:32574287-32574323	ITS	Empty
chr15:23487997-23488047 *	ITS	NF
chr15:29677583-29677653	Empty	Empty
chr15:51951378-51951403	ITS	Empty
chr17:74095846-74095878	ITS	Empty
chr17:24696793-24696817	ITS	Empty
chr17:76046120-76046146	ITS	Empty
chr18:79615942-79615971	ITS	Empty
chr19:32741-32803 *	Empty	Empty
chr19:1124529-1124602	ITS	Empty
chr19:10034261-10034295	Empty	NF
chr20:18458893-18458969	Empty	Empty
chr21:15781540-15781563	ITS	Empty
chr21:52191569-52191606	ITS	Empty
chr22:8731063-8731104	ITS	Empty
chr23:37412257-37412345	ITS	Empty
chr23:40710560-40710607	Empty	Empty
chr23:44283331-44283381	ITS	Empty
chr23:45515290-45515320	ITS	Empty
chr24:26535796-26535829	ITS	Empty
chr25:15560895-15560949	ITS	Empty
chr26:28375514-28375542	ITS	Empty
chr27:11505874-11505908	ITS	Empty
chr27:21217687-21217745 *	Empty	NF
chr27:24213772-24213798	ITS	Empty
chr27:29189054-29189097	ITS	Empty
chr28:18062208-18062233	ITS	Empty
chr28:41719878-41719912	ITS	Empty
chr29:23755261-23755308	ITS	Empty
chr30:11679429-11679454	ITS	Empty
chr30:25048051-25048089	ITS	Empty
chrX:95073117-95073140	ITS	Empty
chrX:118861332-118861368	Empty	Empty

* ITS locus heterozygous in Twilight.

**Table 5:** Flanking sequence modification.

Flanking Sequence Modification	Number of Loci (%)
No modification	11 (16.7)
Deletion	20 (30.3)
Sequence insertion (Total)	13 (19.7)
Random sequence	12 (18.2)
TERC sequence	1 (1.5)
Duplication	3 (4.5)
Complex modification (Total)	19 (28.8)
Deletions and additions	14 (21.2)
Deletion, addition, duplication	2 (3.0)
Complex modifications including inversions	3 (4.5)
**Total**	66 (100)

**Table 6:** Number of loci containing nucleotides in register with the telomeric insertion.

No. of Nucleotides in Register with Telomeric Insertion	Number of Observed Loci (%)	Expected Loci (%)
0	9 (22.50)	(75)
1 or more	31 (77.50)	(≤25)
2 or more	24 (60.00)	(≤6.25)
3 or more	14 (35.00)	(≤1.56)
4 or more	6 (15.00)	(≤0.39)
5 or more	2 (5.00)	(≤0.16)

## Supplementary Materials

The following are available online at https://www.mdpi.com/1422-0067/21/8/2838/s1, Supplementary Table S1. List of human ITS loci ranked by coordinate; Supplementary Table S2. Primer pairs used to PCR-amplify human-specific ITS loci; Supplementary Table S3. List of horse ITS loci ranked by coordinate; Supplementary Table S4. Primer pairs used to PCR amplify horse ITS loci; Supplementary Table S5. Chromosomal distribution of human ITS loci; Supplementary Table S6. Chromosomal distribution of horse ITS loci; Supplementary Figure S1. Search of ITS-less alleles in Twilight.

## Abbreviations

BLAST	Basic Local Alignment Search Tool
BLAT	BLAST-Like Alignment Tool
CAD	carbamyl-P-synthetase, aspartate transcarbamilase, dihydro-orotase
DSB	Double-Strand Break
ERE1	Equine Repetitive Element 1
FISH	Fluorescence In Situ Hybridization
Het-ITS	Heterochromatic ITS
ITS	Interstitial Telomeric Sequence
Numt	Nuclear sequences of mitochondrial origin
PALA	N-(phosphonacetyl)-L-aspartate
PCR	Polymerase Chain Reaction
TERC	Telomerase RNA Component
TERRA	Telomeric Repeat-containing RNA
TRF1	Telomeric Repeat Factor 1
TRF2	Telomeric Repeat Factor 2
VNTR	Variable Number of Tandem Repeats

## References

[1] Shay J.W., Wright W.E. (2019). Telomeres and telomerase: Three decades of progress. Nat. Rev. Genet..

[2] de Lange T. (2018). Shelterin-Mediated Telomere Protection. Annu. Rev. Genet..

[3] Campisi J. (2001). Cellular senescence as a tumor-suppressor mechanism. Trends Cell Biol..

[4] Deng Y., Chan S.S., Chang S. (2008). Telomere dysfunction and tumour suppression: The senescence connection. Nat. Rev. Cancer.

[5] McHugh D., Gil J. (2018). Senescence and aging: Causes, consequences, and therapeutic avenues. J. Cell Biol..

[6] Shay J.W. (2018). Telomeres and aging. Curr. Opin. Cell Biol..

[7] Azzalin C.M., Reichenbach P., Khoriauli L., Giulotto E., Lingner J. (2007). Telomeric repeat containing RNA and RNA surveillance factors at mammalian chromosome ends. Science.

[8] Bettin N., Oss Pegorar C., Cusanelli E. (2019). The Emerging Roles of TERRA in Telomere Maintenance and Genome Stability. Cells.

[9] Schoeftner S., Blasco M.A. (2008). Developmentally regulated transcription of mammalian telomeres by DNA-dependent RNA polymerase II. Nat. Cell Biol..

[10] Vitelli V., Falvo P., G Nergadze S., Santagostino M., Khoriauli L., Pellanda P., Bertino G., Occhini A., Benazzo M., Morbini P. (2018). Telomeric Repeat-Containing RNAs (TERRA) Decrease in Squamous Cell Carcinoma of the Head and Neck Is Associated with Worsened Clinical Outcome. Int. J. Mol. Sci..

[11] Storti C.B., de Oliveira R.A., de Carvalho M., Hasimoto E.N., Cataneo D.C., Cataneo A.J.M., De Faveri J., Vasconcelos E.J.R., Dos Reis P.P., Cano M.I.N. (2019). Telomere-associated genes and telomeric lncRNAs are biomarker candidates in lung squamous cell carcinoma (LUSC). Exp. Mol. Pathol..

[12] Meyne J., Baker R.J., Hobart H.H., Hsu T.C., Ryder O.A., Ward O.G., Wiley J.E., Wurster-Hill D.H., Yates T.L., Moyzis R.K. (1990). Distribution of non-telomeric sites of the (TTAGGG)n telomeric sequence in vertebrate chromosomes. Chromosoma.

[13] Ruiz-Herrera A., Nergadze S.G., Santagostino M., Giulotto E. (2008). Telomeric repeats far from the ends: Mechanisms of origin and role in evolution. Cytogenet. Genome Res..

[14] Faravelli M., Moralli D., Bertoni L., Attolini C., Chernova O., Raimondi E., Giulotto E. (1998). Two extended arrays of a satellite DNA sequence at the centromere and at the short-arm telomere of Chinese hamster chromosome *Cytogenet*. Cell Genet..

[15] Faravelli M., Azzalin C.M., Bertoni L., Chernova O., Attolini C., Mondello C., Giulotto E. (2002). Molecular organization of internal telomeric sequences in Chinese hamster chromosomes. Gene.

[16] Nanda I., Schrama D., Feichtinger W., Haaf T., Schartl M., Schmid M. (2002). Distribution of telomeric (TTAGGG)(n) sequences in avian chromosomes. Chromosoma.

[17] Ventura K., Silva M.J.J., Fagundes V., Christoff A.U., Yonenaga-Yassuda Y. (2006). Non-telomeric sites as evidence of chromosomal rearrangement and repetitive (TTAGGG)n arrays in heterochromatic and euchromatic regions in four species of Akodon (Rodentia, Muridae). Cytogenet. Genome Res..

[18] Rovatsos M., Kratochvíl L., Altmanová M., Johnson Pokorná M. (2015). Interstitial Telomeric Motifs in Squamate Reptiles: When the Exceptions Outnumber the Rule. PLoS ONE.

[19] Zattera M.L., Lima L., Duarte I., de Sousa D.Y., Araújo O.G.D.S., Gazoni T., Mott T., Recco-Pimentel S.M., Bruschi D.P. (2019). Chromosome spreading of the (TTAGGG)n repeats in the Pipa carvalhoi Miranda-Ribeiro, 1937 (Pipidae, Anura) karyotype. Comp. Cytogenet..

[20] López-Fernández C., Arroyo F., Fernández J.L., Gosálvez J. (2006). Interstitial telomeric sequence blocks in constitutive pericentromeric heterochromatin from Pyrgomorpha conica (Orthoptera) are enriched in constitutive alkali-labile sites. Mutat. Res..

[21] He L., Liu J., Torres G.A., Zhang H., Jiang J., Xie C. (2013). Interstitial telomeric repeats are enriched in the centromeres of chromosomes in Solanum species. Chromosome Res..

[22] IJdo J.W., Baldini A., Ward D.C., Reeders S.T., Wells R.A. (1991). Origin of human chromosome 2: An ancestral telomere-telomere fusion. Proc. Natl. Acad. Sci. USA.

[23] Azzalin C.M., Nergadze S.G., Giulotto E. (2001). Human intrachromosomal telomeric-like repeats: Sequence organization and mechanisms of origin. Chromosoma.

[24] Azzalin C.M., Mucciolo E., Bertoni L., Giulotto E. (1997). Fluorescence in situ hybridization with a synthetic (T2AG3)n polynucleotide detects several intrachromosomal telomere-like repeats on human chromosomes. Cytogenet. Cell Genet..

[25] Nergadze S.G., Santagostino M.A., Salzano A., Mondello C., Giulotto E. (2007). Contribution of telomerase RNA retrotranscription to DNA double-strand break repair during mammalian genome evolution. Genome Biol..

[26] Ruiz-Herrera A., García F., Azzalin C., Giulotto E., Egozcue J., Ponsà M., Garcia M. (2002). Distribution of intrachromosomal telomeric sequences (ITS) on Macaca fascicularis (Primates) chromosomes and their implication for chromosome evolution. Hum. Genet..

[27] Nergadze S.G., Rocchi M., Azzalin C.M., Mondello C., Giulotto E. (2004). Insertion of telomeric repeats at intrachromosomal break sites during primate evolution. Genome Res..

[28] Ruiz-Herrera A., García F., Giulotto E., Attolini C., Egozcue J., Ponsà M., Garcia M. (2005). Evolutionary breakpoints are co-localized with fragile sites and intrachromosomal telomeric sequences in primates. Cytogenet. Genome Res..

[29] Bertoni L., Attolini C., Faravelli M., Simi S., Giulotto E. (1996). Intrachromosomal telomere-like DNA sequences in Chinese hamster. Mamm. Genome.

[30] Simi S., Attolini C., Giulotto E. (1998). Intrachromosomal telomeric repeats and stabilization of truncated chromosomes in V79 Chinese hamster cells. Mutat. Res..

[31] de la Seña C., Chowdhary B.P., Gustavsson I. (1995). Localization of the telomeric (TTAGGG)n sequences in chromosomes of some domestic animals by fluorescence in situ hybridization. Hereditas.

[32] Raudsepp T., Christensen K., Chowdhar B.P. (2000). Cytogenetics of donkey chromosomes: Nomenclature proposal based on GTG-banded chromosomes and depiction of NORs and telomeric sites. Chromosome Res..

[33] Santani A., Raudsepp T., Chowdhary B.P. (2002). Interstitial telomeric sites and NORs in Hartmann’s zebra (Equus zebra hartmannae) chromosomes. Chromosome Res..

[34] Schlötterer C., Tautz D. (1992). Slippage synthesis of simple sequence DNA. Nucleic Acids Res..

[35] Camats N., Ruiz-Herrera A., Parrilla J.J., Acien M., Payá P., Giulotto E., Egozcue J., García F., Garcia M. (2006). Genomic instability in rat: Breakpoints induced by ionising radiation and interstitial telomeric-like sequences. Mutat. Res..

[36] Carroll M.L., Roy-Engel A.M., Nguyen S.V., Salem A.H., Vogel E., Vincent B., Myers J., Ahmad Z., Nguyen L., Sammarco M. (2001). Large-scale analysis of the Alu Ya5 and Yb8 subfamilies and their contribution to human genomic diversity. J. Mol. Biol..

[37] Batzer M.A., Deininger P.L. (2002). Alu repeats and human genomic diversity. Nat. Rev. Genet..

[38] Roy-Engel A.M., Salem A.-H., Oyeniran O.O., Deininger L., Hedges D.J., Kilroy G.E., Batzer M.A., Deininger P.L. (2002). Active Alu element “A-tails”: Size does matter. Genome Res..

[39] Salem A.-H., Ray D.A., Xing J., Callinan P.A., Myers J.S., Hedges D.J., Garber R.K., Witherspoon D.J., Jorde L.B., Batzer M.A. (2003). Alu elements and hominid phylogenetics. Proc. Natl. Acad. Sci. USA.

[40] Bennett E.A., Coleman L.E., Tsui C., Pittard W.S., Devine S.E. (2004). Natural genetic variation caused by transposable elements in humans. Genetics.

[41] Wang J., Song L., Gonder M.K., Azrak S., Ray D.A., Batzer M.A., Tishkoff S.A., Liang P. (2006). Whole genome computational comparative genomics: A fruitful approach for ascertaining Alu insertion polymorphisms. Gene.

[42] Walker J.A., Jordan V.E., Storer J.M., Steely C.J., Gonzalez-Quiroga P., Beckstrom T.O., Rewerts L.C., St Romain C.P., Rockwell C.E., Rogers J. (2019). Alu insertion polymorphisms shared by Papio baboons and Theropithecus gelada reveal an intertwined common ancestry. Mob. DNA.

[43] Wang L., Rishishwar L., Mariño-Ramírez L., Jordan I.K. (2017). Human population-specific gene expression and transcriptional network modification with polymorphic transposable elements. Nucleic Acids Res..

[44] Pitkänen E., Cajuso T., Katainen R., Kaasinen E., Välimäki N., Palin K., Taipale J., Aaltonen L.A., Kilpivaara O. (2014). Frequent L1 retrotranspositions originating from TTC28 in colorectal cancer. Oncotarget.

[45] Payer L.M., Steranka J.P., Yang W.R., Kryatova M., Medabalimi S., Ardeljan D., Liu C., Boeke J.D., Avramopoulos D., Burns K.H. (2017). Structural variants caused by Alu insertions are associated with risks for many human diseases. Proc. Natl. Acad. Sci. USA.

[46] Witherspoon D.J., Zhang Y., Xing J., Watkins W.S., Ha H., Batzer M.A., Jorde L.B. (2013). Mobile element scanning (ME-Scan) identifies thousands of novel Alu insertions in diverse human populations. Genome Res..

[47] Steely C.J., Walker J.A., Jordan V.E., Beckstrom T.O., McDaniel C.L., St Romain C.P., Bennett E.C., Robichaux A., Clement B.N., Raveendran M. (2017). Alu Insertion Polymorphisms as Evidence for Population Structure in Baboons. Genome Biol. Evol..

[48] Yu Q., Zhang W., Zhang X., Zeng Y., Wang Y., Wang Y., Xu L., Huang X., Li N., Zhou X. (2017). Population-wide sampling of retrotransposon insertion polymorphisms using deep sequencing and efficient detection. Gigascience.

[49] Santagostino M., Khoriauli L., Gamba R., Bonuglia M., Klipstein O., Piras F.M., Vella F., Russo A., Badiale C., Mazzagatti A. (2015). Genome-wide evolutionary and functional analysis of the Equine Repetitive Element 1: An insertion in the myostatin promoter affects gene expression. BMC Genet..

[50] Nergadze S.G., Lupotto M., Pellanda P., Santagostino M., Vitelli V., Giulotto E. (2010). Mitochondrial DNA insertions in the nuclear horse genome. Anim. Genet..

[51] Trifonov V.A., Stanyon R., Nesterenko A.I., Fu B., Perelman P.L., O’Brien P.C.M., Stone G., Rubtsova N.V., Houck M.L., Robinson T.J. (2008). Multidirectional cross-species painting illuminates the history of karyotypic evolution in Perissodactyla. Chromosome Res..

[52] Wade C.M., Giulotto E., Sigurdsson S., Zoli M., Gnerre S., Imsland F., Lear T.L., Adelson D.L., Bailey E., Bellone R.R. (2009). Genome sequence, comparative analysis, and population genetics of the domestic horse. Science.

[53] Piras F.M., Nergadze S.G., Magnani E., Bertoni L., Attolini C., Khoriauli L., Raimondi E., Giulotto E. (2010). Uncoupling of satellite DNA and centromeric function in the genus Equus. PLoS Genet..

[54] Steiner C.C., Ryder O.A. (2011). Molecular phylogeny and evolution of the Perissodactyla. Zool. J. Linn. Soc..

[55] Orlando L., Ginolhac A., Zhang G., Froese D., Albrechtsen A., Stiller M., Schubert M., Cappellini E., Petersen B., Moltke I. (2013). Recalibrating Equus evolution using the genome sequence of an early Middle Pleistocene horse. Nature.

[56] Jónsson H., Schubert M., Seguin-Orlando A., Ginolhac A., Petersen L., Fumagalli M., Albrechtsen A., Petersen B., Korneliussen T.S., Vilstrup J.T. (2014). Speciation with gene flow in equids despite extensive chromosomal plasticity. Proc. Natl. Acad. Sci. USA.

[57] Giulotto E., Raimondi E., Sullivan K.F. (2017). The Unique DNA Sequences Underlying Equine Centromeres. Prog. Mol. Subcell. Biol..

[58] Nergadze S.G., Piras F.M., Gamba R., Corbo M., Cerutti F., McCarter J.G.W., Cappelletti E., Gozzo F., Harman R.M., Antczak D.F. (2018). Birth, evolution, and transmission of satellite-free mammalian centromeric domains. Genome Res..

[59] Sudmant P.H., Rausch T., Gardner E.J., Handsaker R.E., Abyzov A., Huddleston J., Zhang Y., Ye K., Jun G., Fritz M.H.-Y. (2015). An integrated map of structural variation in 2,504 human genomes. Nature.

[60] Bertoni L., Attolini C., Tessera L., Mucciolo E., Giulotto E. (1994). Telomeric and nontelomeric (TTAGGG)n sequences in gene amplification and chromosome stability. Genomics.

[61] Kalbfleisch T.S., Rice E.S., DePriest M.S., Walenz B.P., Hestand M.S., Vermeesch J.R., O Connell B.L., Fiddes I.T., Vershinina A.O., Saremi N.F. (2018). Improved reference genome for the domestic horse increases assembly contiguity and composition. Commun. Biol..

[62] BLAST NCBI Trace Database. https://blast.ncbi.nlm.nih.gov/Blast.cgi?PAGE=Nucleotides&PROGRAM=blastn&BLAST_SPEC=TraceArchive&BLAST_PROGRAMS=megaBlast&PAGE_TYPE=BlastSearch.

[63] Mondello C., Pirzio L., Azzalin C.M., Giulotto E. (2000). Instability of interstitial telomeric sequences in the human genome. Genomics.

[64] Huang J., Zhao Y., Bai D., Shiraigol W., Li B., Yang L., Wu J., Bao W., Ren X., Jin B. (2015). Donkey genome and insight into the imprinting of fast karyotype evolution. Sci. Rep..

[65] Renaud G., Petersen B., Seguin-Orlando A., Bertelsen M.F., Waller A., Newton R., Paillot R., Bryant N., Vaudin M., Librado P. (2018). Improved de novo genomic assembly for the domestic donkey. Sci. Adv..

[66] Equus Asinus Assembly ASM130575v1, Breed: Guanzhong Donkey. https://www.ncbi.nlm.nih.gov/assembly/GCF_001305755.1.

[67] Equus asinus asinus assembly ASM303372v1, Willy. https://www.ncbi.nlm.nih.gov/assembly/GCA_003033725.1.

[68] Ceratotherium simum simum assembly CerSimSim1.0. https://www.ncbi.nlm.nih.gov/assembly/GCF_000283155.1/.

[69] Simonet T., Zaragosi L.-E., Philippe C., Lebrigand K., Schouteden C., Augereau A., Bauwens S., Ye J., Santagostino M., Giulotto E. (2011). The human TTAGGG repeat factors 1 and 2 bind to a subset of interstitial telomeric sequences and satellite repeats. Cell Res..

[70] Murphy W.J., Pringle T.H., Crider T.A., Springer M.S., Miller W. (2007). Using genomic data to unravel the root of the placental mammal phylogeny. Genome Res..

[71] Lander E.S., Linton L.M., Birren B., Nusbaum C., Zody M.C., Baldwin J., Devon K., Dewar K., Doyle M., FitzHugh W. (2001). Initial sequencing and analysis of the human genome. Nature.

[72] Rosato M., Álvarez I., Feliner G.N., Rosselló J.A. (2018). Inter- and intraspecific hypervariability in interstitial telomeric-like repeats (TTTAGGG)n in Anacyclus (Asteraceae). Ann. Bot..

[73] Wakefield S., Knowles J., Zimmermann W., van Dierendonck M., IUCN/SSC Equid Specialist Group, International Union for Conservation of Nature and Natural Resources (2002). Chapter 7: Status and action plan for the Przewalski’s horse (equus ferus przewalskii). Equids-Zebras, Asses, and Horses: Status Survey and Conservation Action Plan.

[74] Rouet P., Smih F., Jasin M. (1994). Introduction of double-strand breaks into the genome of mouse cells by expression of a rare-cutting endonuclease. Mol. Cell. Biol..

[75] Rebuzzini P., Khoriauli L., Azzalin C.M., Magnani E., Mondello C., Giulotto E. (2005). New mammalian cellular systems to study mutations introduced at the break site by non-homologous end-joining. DNA Repair (Amst.).

[76] Onozawa M., Zhang Z., Kim Y.J., Goldberg L., Varga T., Bergsagel P.L., Kuehl W.M., Aplan P.D. (2014). Repair of DNA double-strand breaks by templated nucleotide sequence insertions derived from distant regions of the genome. Proc. Natl. Acad. Sci. USA.

[77] Chang H.H.Y., Pannunzio N.R., Adachi N., Lieber M.R. (2017). Non-homologous DNA end joining and alternative pathways to double-strand break repair. Nat. Rev. Mol. Cell Biol..

[78] Ségal-Bendirdjian E., Geli V. (2019). Non-canonical Roles of Telomerase: Unraveling the Imbroglio. Front. Cell Dev. Biol..

[79] Homo Sapiens (human) Nucleotide BLAST. https://blast.ncbi.nlm.nih.gov/Blast.cgi?PAGE_TYPE=BlastSearch&BLAST_SPEC=OGP__9606__9558&LINK_LOC=blasthome.

[80] Galaxy. https://usegalaxy.org/.

[81] Afgan E., Baker D., van den Beek M., Blankenberg D., Bouvier D., Čech M., Chilton J., Clements D., Coraor N., Eberhard C. (2016). The Galaxy platform for accessible, reproducible and collaborative biomedical analyses: 2016 update. Nucleic Acids Res..

[82] UCSC Genome Browser, Track “1000 Genomes Phase 3 Integrated Variant Calls: SNVs, Indels, SVs”. https://genome.ucsc.edu/cgi-bin/hgTrackUi?hgsid=720970681_qqmhEowWab8OoZ9mluPovptBdLXW&c=chrX&g=tgpPhase3.

[83] Semino O., Magri C., Benuzzi G., Lin A.A., Al-Zahery N., Battaglia V., Maccioni L., Triantaphyllidis C., Shen P., Oefner P.J. (2004). Origin, diffusion, and differentiation of Y-chromosome haplogroups E and J: Inferences on the neolithization of Europe and later migratory events in the Mediterranean area. Am. J. Hum. Genet..

[84] UCSC Genome Browser Gateway. https://genome.ucsc.edu/cgi-bin/hgGateway.

[85] Anglana M., Bertoni L., Giulotto E. (1996). Cloning of a polymorphic sequence from the nontranscribed spacer of horse rDNA. Mamm. Genome.

[86] Multalin. http://multalin.toulouse.inra.fr/multalin/.

[87] Corpet F. (1988). Multiple sequence alignment with hierarchical clustering. Nucleic Acids Res..

[88] Vidale P., Magnani E., Nergadze S.G., Santagostino M., Cristofari G., Smirnova A., Mondello C., Giulotto E. (2012). The catalytic and the RNA subunits of human telomerase are required to immortalize equid primary fibroblasts. Chromosoma.

[89] Piras F.M., Nergadze S.G., Poletto V., Cerutti F., Ryder O.A., Leeb T., Raimondi E., Giulotto E. (2009). Phylogeny of Horse Chromosome 5q in the Genus Equus and Centromere Repositioning. Cytogenet. Genome Res..

