# Supplementary material for: Insertion of Telomeric Repeats in the Human and Horse Genomes: An Evolutionary Perspective
Source: Int J Mol Sci. 2020 Apr 18;21(8):2838. doi: 10.3390/ijms21082838 (PMC7215372; doi:10.3390/ijms21082838)
Supplement: Supplementary file 1 [file ijms-21-02838-s001.pdf]

Supplementary Table S1. List of human ITS loci ranked by coordinate.

Number	Locus	ITS length (mismatches)	Number of mismatch/repeat
1	chr1:5225322-5225352	31 (2)	0.39
2	chr1:11500152-11500219	68 (8)	0.71
3	chr1:12684777-12684817	41 (4)	0.59
4	chr1:18580852-18580885	34 (0)	0.00
5	chr1:41747615-41747658	44 (2)	0.27
6	chr1:57237280-57237309	30 (3)	0.60
7	chr1:78594426-78594461	36 (0)	0.00
8	chr1:79835766-79835789	24 (1)	0.25
9	chr1:98444328-98444364	37 (5)	0.81
10	chr1:110646800-110646825	26 (2)	0.46
11	chr1:182644338-182644363	26 (2)	0.46
12	chr1:226653352-226653399	48 (4)	0.50
13	chr2:7494201-7494226	26 (3)	0.69
14	chr2:11146521-11146552	32 (3)	0.56
15	chr2:50067529-50067562	34 (4)	0.71
16	chr2:75299632-75299673	42 (4)	0.57
17	chr2:75756308-75756333	26 (2)	0.46
18	chr2:87142399-87142428	28 (1)	0.21
19	chr2:114360253-114361060	808 (104)	0.77
20	chr2:114382097-114382307	211 (27)	0.77
21	chr2:122226335-122226364	30 (4)	0.80
22	chr2:122916840-122916894	55 (8)	0.87
23	chr2:124888343-124888366	24 (3)	0.75
24	chr2:132560176-132560201	26 (3)	0.69
25	chr2:145516467-145516492	26 (3)	0.69
26	chr2:182140519-182140585	67 (0)	0.00
27	chr2:220568402-220568438	37 (4)	0.65
28	chr2:222928086-222928178	93 (4)	0.26
29	chr2:228700293-228700331	39 (5)	0.77
30	chr2:229979859-229979887	29 (3)	0.62
31	chr2:232110183-232110214	32 (4)	0.75
32	chr2:233267721-233267757	37 (3)	0.49
33	chr2:233319863-233319887	25 (1)	0.24
34	chr2:241161288-241161322	35 (0)	0.00
35	chr2:241552916-241553771	856 (133)	0.93
36	chr3:44383913-44383936	24 (3)	0.75
37	chr3:50808183-50808238	56 (5)	0.54
38	chr3:59407504-59407528	25 (0)	0.00
39	chr3:113121460-113121483	24 (3)	0.75
40	chr3:123153676-123153701	26 (1)	0.23
41	chr3:134165409-134165438	30 (3)	0.60
42	chr3:134396458-134396494	37 (3)	0.49
43*	chr3:159412014-159412054	41 (1)	0.15
44	chr3:181540026-181540059	34 (3)	0.53
45	chr3:197899573-197901399	1826 (245)	0.81
46	chr4:698746-699213	468 (78)	1.00
47	chr4:6425629-6425664	36 (2)	0.33
48	chr4:8349085-8349134	50 (5)	0.60
49	chr4:47206503-47206533	31 (2)	0.39

50	chr4:107711621-107711644	24 (1)	0.25
51	chr4:191024114-191024141	28 (1)	0.21
52	chr5:49637-49904	268 (39)	0.87
53	chr5:1154291-1154342	52 (4)	0.46
54	chr5:2185125-2185319	195 (25)	0.77
55	chr5:2999610-2999671	62 (10)	0.97
56	chr5:16348176-16348232	57 (8)	0.84
57	chr5:17987270-17987300	31 (4)	0.77
58	chr5:40729066-40729094	29 (0)	0.00
59	chr5:66838592-66838622	31 (3)	0.58
60	chr5:82811939-82811979	41 (2)	0.29
61	chr5:88629411-88629451	41 (2)	0.29
62	chr5:151219197-151219229	33 (3)	0.55
63	chr5:166368107-166368132	26 (1)	0.23
64	chr5:168340203-168340228	26 (2)	0.46
65	chr6:147636-147986	351 (33)	0.56
66	chr6:1114985-1115028	44 (2)	0.27
67	chr6:6926059-6926098	40 (5)	0.75
68	chr6:12067355-12067388	34 (3)	0.53
69	chr6:38912724-38912804	81 (5)	0.37
70	chr6:116072681-116072710	30 (3)	0.60
71	chr6:134541766-134541796	31 (3)	0.58
72	chr6:135875505-135875560	56 (9)	0.96
73	chr6:168213110-168213142	33 (2)	0.36
74	chr7:16384-16986	603 (77)	0.77
75	chr7:173855-173884	30 (0)	0.00
76	chr7:1228277-1228317	41 (3)	0.44
77	chr7:1795896-1796103	208 (28)	0.81
78	chr7:31400803-31400832	30 (4)	0.80
79	chr7:41163791-41163817	27 (2)	0.44
80	chr7:44137004-44137037	34 (2)	0.35
81	chr7:66817887-66817930	44 (7)	0.95
82	chr7:71885143-71885179	37 (4)	0.65
83	chr7:85236694-85236752	58 (7)	0.72
84	chr7:152595062-152595136	75 (11)	0.88
85	chr7:155564958-155565005	48 (3)	0.38
86	chr7:155978696-155978723	28 (1)	0.21
87	chr8:155248-155736	489 (57)	0.70
88	chr8:170438-170596	159 (19)	0.72
89	chr8:513059-513093	35 (5)	0.86
90	chr8:35465356-35465394	39 (1)	0.15
91	chr8:37094171-37094198	28 (1)	0.21
92	chr8:66813911-66813941	31 (2)	0.39
93	chr8:76901542-76901570	29 (2)	0.41
94	chr8:97810860-97810883	24 (2)	0.50
95	chr8:113187594-113187622	29 (3)	0.62
96	chr8:113722034-113722059	26 (1)	0.23
97	chr8:115858454-115858479	26 (2)	0.46
98	chr8:125816674-125816711	38 (4)	0.63
99	chr8:144268095-144268130	36 (3)	0.50
100	chr9:2824045-2824081	37 (0)	0.00
101	chr9:13958230-13958253	24 (0)	0.00

102	chr9:34716247-34716271	25 (2)	0.48
103	chr9:71880154-71880224	71 (3)	0.25
104	chr9:130916547-130916604	58 (1)	0.10
105	chr9:132927632-132927731	100 (12)	0.72
106	chr9:137102218-137102251	34 (1)	0.18
107	chr9:141009614-141009638	25 (3)	0.72
108	chr9:141023514-141023665	152 (18)	0.71
109	chr9:141127099-141127856	758 (104)	0.82
110	chr10:110213-110551	339 (48)	0.85
111	chr10:3576258-3576289	32 (3)	0.56
112	chr10:3986187-3986375	189 (27)	0.86
113	chr10:4111271-4111429	159 (26)	0.98
114	chr10:8510395-8510514	120 (11)	0.55
115	chr10:25696846-25696869	24 (1)	0.25
116	chr10:26833346-26833369	24 (1)	0.25
117	chr10:47666808-47666939	132 (9)	0.41
118	chr10:47667355-47667385	31 (0)	0.00
119	chr10:48334148-48334286	139 (10)	0.43
120	chr10:78027933-78027957	25 (1)	0.24
121	chr10:80634992-80635043	52 (6)	0.69
122	chr10:81302983-81303009	27 (2)	0.44
123	chr10:81976698-81976735	38 (4)	0.63
124	chr10:95545535-95545561	41 (4)	0.59
125	chr10:126751097-126751120	24 (2)	0.50
126	chr10:135502708-135503008	301 (40)	0.80
127	chr11:175280-176542	1263 (141)	0.67
128	chr11:191800-192014	215 (19)	0.53
129	chr11:1867061-1867092	32 (2)	0.38
130	chr11:2145474-2145498	25 (1)	0.24
131	chr11:10686674-10686700	27 (0)	0.00
132	chr11:25547748-25547771	24 (0)	0.00
133	chr11:62795667-62795721	55 (3)	0.33
134	chr11:69083922-69083949	28 (1)	0.21
135	chr11:80597886-80598032	147 (18)	0.73
136	chr11:87636289-87636312	24 (0)	0.00
137	chr11:124403484-124403520	37 (2)	0.32
138*	chr11:129573442-129573473	32 (1)	0.19
139	chr12:95155-95739	585 (28)	0.29
140	chr12:217258-217292	35 (0)	0.00
141	chr12:15500986-15501009	24 (3)	0.75
142	chr12:16156475-16156499	25 (4)	0.96
143	chr12:22393976-22394010	35 (1)	0.17
144	chr12:98708652-98708677	26 (1)	0.23
145	chr12:108411954-108411977	24 (3)	0.75
146	chr12:112972346-112972393	48 (5)	0.63
147	chr12:121634448-121634510	64 (8)	0.75
148	chr12:124751892-124752088	197 (27)	0.82
149	chr12:127049926-127049999	74 (9)	0.73
150	chr13:82281192-82281220	29 (3)	0.62
151	chr13:91357864-91357908	45 (3)	0.40
152	chr13:111116610-111116644	35 (4)	0.69
153	chr13:112322118-112322158	41 (4)	0.59

154	chr13:112653749-112653946	198 (31)	0.94
155	chr13:113558442-113558558	117 (19)	0.97
156	chr13:114979498-114979524	27 (0)	0.00
157	chr14:19349224-19349553	330 (52)	0.95
158	chr14:49573301-49573325	25 (2)	0.48
159	chr14:78895373-78895400	28 (3)	0.64
160	chr14:99843383-99843409	27 (2)	0.44
161	chr14:101057046-101057118	73 (11)	0.90
162*	chr15:42243201-42243234	34 (0)	0.00
163	chr15:71291951-71291978	28 (2)	0.43
164	chr15:80755813-80755843	31 (0)	0.00
165	chr16:69530-69889	360 (57)	0.95
166	chr16:790561-790584	24 (3)	0.75
167	chr16:10346945-10346975	31 (1)	0.19
168	chr16:13937719-13937747	29 (4)	0.83
169	chr16:25771417-25771480	64 (4)	0.38
170	chr16:29263664-29263689	26 (3)	0.69
171	chr16:49817125-49817559	435 (31)	0.43
172	chr16:65278091-65278136	46 (4)	0.52
173*	chr16:75368098-75368138	41 (0)	0.00
174	chr16:76150958-76151012	55 (6)	0.65
175	chr16:76175694-76175724	31 (2)	0.39
176	chr16:81956779-81956864	86 (10)	0.70
177	chr16:88258578-88258603	26 (3)	0.69
178	chr16:88946631-88946670	40 (3)	0.45
179	chr16:90188062-90188814	753 (98)	0.78
180	chr17:11313813-11313837	25 (3)	0.72
181	chr17:12375833-12375857	25 (2)	0.48
182	chr17:37853388-37853413	25 (3)	0.72
183	chr17:45781987-45782014	28 (3)	0.64
184	chr17:76468335-76468406	72 (12)	1.00
185	chr18:63615-64200	586 (85)	0.87
186	chr18:94514-94691	178 (23)	0.78
187	chr18:98752-98793	42 (2)	0.29
188	chr18:105193-105670	478 (55)	0.69
189	chr18:61642812-61642836	25 (1)	0.24
190	chr18:78016174-78016404	231 (35)	0.91
191	chr19:245537-246002	466 (56)	0.72
192	chr19:7932254-7932277	24 (1)	0.25
193	chr19:16765839-16765884	46 (6)	0.78
194	chr19:17666248-17666277	30 (5)	1.00
195	chr19:22189254-22189292	39 (5)	0.77
196	chr19:38129415-38129444	30 (4)	0.80
197	chr19:53230794-53230818	25 (3)	0.72
198	chr19:59092324-59092348	25 (2)	0.48
199	chr19:59097932-59098077	146 (11)	0.45
200	chr20:10172183-10172217	35 (2)	0.34
201	chr20:19717923-19717948	26 (2)	0.46
202	chr20:33051237-33051260	24 (0)	0.00
203	chr20:43941919-43942102	184 (21)	0.68
204	chr20:62770975-62771001	27 (2)	0.44
205	chr20:62918053-62918986	934 (94)	0.60

206	chr21:23134968-23135002	35 (3)	0.51
207	chr21:36085146-36085229	84 (6)	0.43
208	chr21:39208108-39208140	33 (0)	0.00
209	chr21:45851809-45851883	75 (8)	0.64
210	chr22:43261817-43261847	31 (0)	0.00
211	chr22:45022395-45022454	60 (4)	0.40
212	chr22:51224560-51224818	259 (35)	0.81
213	chrX:3293289-3293345	57 (6)	0.63
214	chrX:6029043-6029076	34 (2)	0.35
215	chrX:66746096-66746124	29 (2)	0.41
216	chrX:136712827-136712850	24 (2)	0.50
217	chrX:154593437-154593471	35 (1)	0.17
218	chrX:154706753-154706787	35 (1)	0.17
219	chrX:155181604-155181649	46 (7)	0.91
220	chrX:155210099-155210174	76 (11)	0.87
221	chrX:155210613-155210676	64 (10)	0.94
222	chrY:2266354-2266386	33 (5)	0.91
223	chrY:7129088-7129129	42 (3)	0.43
224	chrY:14036287-14036338	52 (5)	0.58
225	chrY:19309207-19309232	26 (1)	0.23
226	chrY:23247861-23247884	24 (2)	0.50
227	chrY:59284610-59284655	46 (7)	0.91
228	chrY:59313619-59313682	64 (9)	0.84
229	chrY:59352521-59352891	371 (62)	1.00

*ITS locus analyzed by PCR.

Supplementary Table S2. Primer pairs used to PCR-amplify human-specific ITS loci. Coordinates of the human-specific ITS loci, oligonucleotide sequences, length of the PCR products for the ITS-containing loci and annealing temperature of each primer pair are indicated.

Locus	Oligonucleotide Sequence	Product Length (bp)	Annealing Temperature (°C)
chr3:159412014-159412054	Forward GCAGAGTTTTGCTCTTATCGG<br>Reverse CAAATCCCATGAGGTATACT	359	60
chr11:129573442-129573473	Forward GTAGGCACAGATGTCCTCTA<br>Reverse TGTCGTGTGCGCTGACCCAAG	250	58
chr15:42243201-42243234	Forward GTTTGGACTGGGAATCACTG<br>Reverse TGGCAACAGTATTGTCTGTG	206	63
chr16:75368098-75368138	Forward CCCAGGAAAGTGCAGATGTT<br>Reverse TCTGTGCAACAGAGGTATGG	353	56

Supplementary Table S3. List of horse ITS loci ranked by coordinate.

Number	Locus	ITS Length (Mismatches)	Number of Mismatch/Repeat
1	chr1:2244304-2244329	26 (2)	0.46
2	chr1:6651108-6651164	57 (8)	0.84
3	chr1:6734830-6734863	34 (5)	0.88
4	chr1:22456681-22456705	25 (3)	0.72
5	chr1:27309501-27309532	32 (3)	0.56
6	chr1:90210027-90210107	81 (11)	0.81
7	chr1:90843617-90843649	33 (0)	0.00
8	chr1:91725039-91725121	83 (13)	0.94
9	chr1:98488870-98488928	59 (8)	0.81

10	chr1:109251859-109251903	45 (3)	0.40
11	chr1:113472802-113472870	69 (8)	0.70
12	chr1:126483145-126483168	24 (3)	0.75
13	chr1:156995264-156995361	98 (9)	0.55
14	chr1:160205664-160205688	25 (3)	0.72
15	chr1:184553488-184553513	26 (1)	0.23
16	chr1:188122300-188122503	204 (32)	0.94
17	chr1:188184755-188184952	198 (30)	0.91
18	chr2:661976-662028	53 (5)	0.57
19*	chr2:13178780-13178780	112 (18)	0.96
20	chr2:19293258-19293287	30 (2)	0.40
21	chr2:30174747-30174793	47 (3)	0.38
22	chr2:48762482-48762505	24 (1)	0.25
23	chr2:48999210-48999725	516 (81)	0.94
24	chr2:49002378-49003329	952 (113)	0.71
25	chr3:6047114-6047139	26 (1)	0.23
26	chr3:45427234-45427258	25 (3)	0.72
27	chr3:61277039-61277063	25 (2)	0.48
28	chr3:68906105-68906142	38 (2)	0.32
29	chr3:82188585-82188664	80 (12)	0.90
30	chr3:92686382-92686405	24 (2)	0.50
31	chr3:106340791-106340815	25 (1)	0.24
32	chr4:14886665-14886705	41 (5)	0.73
33	chr4:21224032-21224087	56 (9)	0.96
34	chr4:65502437-65502468	32 (1)	0.19
35	chr4:68540207-68540247	41 (3)	0.44
36	chr4:73338748-73338783	36 (5)	0.83
37	chr4:81320764-81320806	43 (3)	0.42
38	chr4:90006773-90006813	41 (0)	0.00
39	chr4:90900902-90900926	25 (4)	0.96
40	chr5:16621900-16621940	41 (6)	0.88
41	chr5:18103531-18103568	38 (3)	0.47
42	chr5:36007702-36007752	51 (0)	0.00
43	chr5:39366430-39366468	39 (3)	0.46
44	chr5:59679920-59679946	27 (3)	0.67
45	chr5:88367091-88367115	25 (0)	0.00
46	chr5:90360200-90360234	35 (0)	0.00
47	chr6:186395-186517	123 (3)	0.15
48	chr6:8289417-8289447	31 (1)	0.19
49	chr6:27940702-27941139	438 (53)	0.73
50	chr6:38922364-38922401	38 (3)	0.47
51	chr7:102615-102640	26 (0)	0.00
52	chr7:5171152-5171236	85 (11)	0.78
53	chr7:29297817-29297852	36 (2)	0.33
54	chr7:51713092-51713124	33 (3)	0.55
55	chr7:80018664-80018693	30 (3)	0.60
56	chr7:100658783-100658811	29 (0)	0.00
57	chr7:100764221-100764245	25 (0)	0.00
58	chr8:7301244-7301267	24 (3)	0.75
59	chr8:24251768-24251863	96 (7)	0.44
60	chr8:32734178-32734236	59 (7)	0.71

61	chr8:39145464-39145491	28 (2)	0.43
62	chr8:64629752-64629778	27 (1)	0.22
63	chr8:83332146-83332181	36 (2)	0.33
64	chr8:90045320-90045361	42 (4)	0.57
65	chr8:94527922-94527948	27 (3)	0.67
66	chr8:94624888-94624932	45 (5)	0.67
67	chr9:359615-359862	248 (37)	0.89
68*	chr9:1570127-1570127	172 (11)	0.38
69	chr9:74200299-74200328	30 (0)	0.00
70	chr9:80878399-80878422	24 (1)	0.25
71	chr10:7134943-7135113	171 (28)	0.98
72	chr10:16415722-16415748	27 (3)	0.67
73	chr10:22622806-22622833	28 (0)	0.00
74	chr10:28666897-28668288	1392 (208)	0.90
75	chr10:28673805-28675202	1398 (213)	0.91
76	chr10:81331655-81331689	35 (3)	0.51
77	chr11:4354977-4355000	24 (3)	0.75
78	chr11:18251540-18251570	31 (2)	0.39
79	chr11:42736447-42736474	28 (1)	0.21
80	chr11:44273764-44273793	30 (3)	0.60
81	chr11:44810227-44810253	27 (1)	0.22
82	chr11:50230428-50230463	36 (2)	0.33
83	chr12:13851529-13851563	35 (1)	0.17
84	chr12:30212139-30212164	26 (2)	0.46
85	chr12:32574287-32574323	37 (2)	0.32
86	chr12:32645063-32645088	26 (3)	0.69
87	chr13:17896076-17896106	31 (2)	0.39
88*	chr15:23487997-23488047	51 (0)	0.00
89	chr15:29677583-29677653	71 (0)	0.00
90	chr15:51951378-51951403	26 (0)	0.00
91	chr15:70935037-70935061	25 (1)	0.24
92	chr15:77022476-77022502	27 (0)	0.00
93	chr16:30945221-30945251	31 (2)	0.39
94	chr17:24696793-24696817	25 (1)	0.24
95	chr17:61213552-61213575	24 (1)	0.25
96	chr17:74095846-74095878	33 (1)	0.18
97	chr17:76046120-76046146	27 (0)	0.00
98	chr18:54029538-54029573	36 (3)	0.50
99	chr18:79615942-79615971	30 (1)	0.20
100*	chr19:32741-32803	63 (0)	0.00
101	chr19:1124529-1124602	74 (9)	0.73
102	chr19:10034261-10034295	35 (0)	0.00
103	chr19:32870789-32870812	24 (2)	0.50
104	chr19:36216441-36216472	32 (3)	0.56
105	chr20:18458893-18458969	77 (4)	0.31
106	chr20:65335998-65341663	5666 (404)	0.43
107	chr21:15781540-15781563	24 (1)	0.25
108	chr21:37927218-37927244	27 (2)	0.44
109	chr21:51541559-51541591	33 (3)	0.55
110	chr21:52191569-52191606	38 (2)	0.32
111	chr22:1388418-1388464	47 (2)	0.26

112	chr22:8731063-8731104	42 (0)	0.00
113	chr22:22797769-22797794	26 (2)	0.46
114	chr22:50795087-50795120	34 (5)	0.88
115	chr22:50922666-50922759	94 (8)	0.51
116	chr23:37412257-37412345	89 (9)	0.61
117	chr23:40710560-40710607	48 (0)	0.00
118	chr23:44283331-44283381	51 (1)	0.12
119	chr23:45515290-45515320	31 (2)	0.39
120	chr24:23116976-23117010	35 (2)	0.34
121	chr24:26535796-26535829	34 (3)	0.53
122	chr24:47924179-47926467	2289 (160)	0.42
123	chr25:15560895-15560949	55 (2)	0.22
124	chr25:40164564-40168162	3599 (444)	0.74
125	chr26:28375514-28375542	29 (0)	0.00
126	chr26:32420315-32420368	54 (8)	0.89
127	chr27:1085318-1085355	38 (6)	0.95
128	chr27:11505874-11505908	35 (4)	0.69
129*	chr27:21217687-21217745	59 (1)	0.10
130	chr27:24213772-24213798	27 (1)	0.22
131	chr27:29189054-29189097	44 (3)	0.41
132	chr28:18062208-18062233	26 (1)	0.23
133	chr28:41719878-41719912	35 (2)	0.34
134	chr29:23755261-23755308	48 (1)	0.13
135	chr30:11679429-11679454	26 (0)	0.00
136	chr30:25048051-25048089	39 (3)	0.46
137	chr30:26328378-26328403	26 (3)	0.69
138	chr31:286978-287002	25 (1)	0.24
139	chr31:461189-461215	27 (3)	0.67
140	chr31:6933180-6933203	24 (1)	0.25
141	chrX:95073117-95073140	24 (2)	0.50
142	chrX:118861332-118861368	37 (1)	0.16

*ITS locus heterozygous in Twilight.

Supplementary Table S4. Primer pairs used to PCR amplify horse ITS loci. Coordinates of the horse ITS loci, primer pairs and oligonucleotide sequences, length of the PCR products for the ITS-containing and the empty alleles and annealing temperature of each primer pair are indicated.

		Product Length (bp)		
**Locus**	**Oligonucleotide Sequence**	**ITS-Containing Allele in Twilight**	**Empty Allele in Twilight Genome**	**Annealing Temperature (°C)**
chr5:90360200-90360234	Forward CATCCACCAGGGACTAGAACA<br>Reverse ACGTAAGTGAGTGAGCTGGAGT	323	No empty allele	60
chr8:24251768-24251863	Forward CCTAGTAGCAGCTGGTAATCT<br>Reverse TGAAAATGTACCTGGTTATAGA	185	No empty allele	57
chr19:10034261-10034295	Forward AGAGCTTCCCTGTAGGAAGCA<br>Reverse CCTAATAGCACAGGAATCCAC	289	No empty allele	60
	Forward1 AGCTGTAGCCCTGTTAGAGA<br>Reverse CCTAATAGCACAGGAATCCAC	306	No empty allele	58
chr20:18458893-18458969	Forward CTTCAGGAAGAAGAAGTATGAC<br>Reverse AGTTTGATACCAGATGATATGGA	240	No empty allele	58
chr23:44283331-44283381	Forward TGATAGCCTACTGAACGGAGA<br>Reverse AGAGAGTAACAGCCTCCTGT	350	No empty allele	60
chr25:15560895-15560949	Forward TCTGGTTCCAACTTGCTGTTC<br>Reverse TGGCAATGTCAGCTGGTTGTCA	417	No empty allele	60
chr27:29189054-29189097	Forward TTCACTTACCATCACTTACTATTC<br>Reverse GAGCTGATATGGTTTAAGTTGC	125	No empty allele	58
chr29:23755261-23755308	Forward GCTGTCAGTCTCCGATTGCCT<br>Reverse GATTCCTCTTCTGTGACATCGT	156	No empty allele	61
chr2:13178780-13178780	Forward GAAACAAATTTAGGGAAAGGAA<br>Reverse CCTGGAATGTACATAAGAATGCT	269	157	57
chr15:23487997-23488047	Forward GGAGCTGCATCCTTAATCTGA<br>Reverse CATGGCCACTGACGAGTGGTGT	347	293	58
	Forward1 CCAGAGCTTTAAATCCCTATGC<br>Reverse1 CTGAAGCACAGCATACTGAAC	233	182	60
chr19:32741-32803	Forward GGAAAAGGTATAATGTGCTGA<br>Reverse GCATAGTTATTTAAATATATAGTTATC	158	82	56
	Forward GGAAAAGGTATAATGTGCTGA<br>Reverse1 AACTGAGAATTCCAAAGAGGTA	1106	1030	58

Supplementary Table S5. Chromosomal distribution of human ITS loci.

Chromosome	Length of Assembled Sequences (Mb)	No. of ITSs	No. of ITS/Mb
21	48.129895	4	8.31E-02
22	51.304566	3	5.85E-02
19	59.128983	9	1.52E-01
Y	59.373566	8	1.35E-01
20	63.02552	6	9.52E-02
18	78.077248	6	7.68E-02
17	81.19521	5	6.16E-02
16	90.354753	15	1.66E-01
15	102.531392	3	2.93E-02
14	107.34954	5	4.66E-02
13	115.169878	7	6.08E-02
12	133.851895	11	8.22E-02
11	135.006516	12	8.89E-02
10	135.534747	17	1.25E-01
9	141.213431	10	7.08E-02
8	146.364022	13	8.88E-02
X	155.27056	9	5.80E-02
7	159.138663	13	8.17E-02
6	171.115067	9	5.26E-02
5	180.91526	13	7.19E-02
4	191.154276	6	3.14E-02
3	198.02243	10	5.05E-02
2	243.199373	23	9.46E-02
1	249.250621	12	4.81E-02

Supplementary Table S6. Chromosomal distribution of horse ITS loci.

Chromosome	Length of Assembled Sequences (Mb)	No. of ITSs	No. of ITS/Mb
31	26.001039	3	1.15E-01
30	31.395959	3	9.56E-02
29	34.77612	1	2.88E-02
12	36.992759	4	1.08E-01
27	40.25469	5	1.24E-01
25	40.282968	2	4.96E-02
26	43.147642	2	4.64E-02
13	43.784481	1	2.28E-02
28	47.348498	2	4.22E-02
24	48.288683	3	6.21E-02
22	50.928189	5	9.82E-02
23	55.556184	4	7.20E-02
21	58.984458	4	6.78E-02
11	61.676917	6	9.73E-02
19	62.681739	5	7.98E-02
20	65.343332	2	3.06E-02
17	80.72243	4	4.96E-02

18	82.641348	2	2.42E-02
10	85.155674	6	7.05E-02
9	85.793548	4	4.66E-02
6	87.230776	4	4.59E-02
16	88.962352	1	1.12E-02
15	92.851403	5	5.38E-02
14	94.600235	0	0.00E+00
5	96.759418	7	7.23E-02
8	97.563019	9	9.22E-02
7	100.787686	7	6.95E-02
4	109.462549	8	7.31E-02
2	121.350024	7	5.77E-02
3	121.351753	7	5.77E-02
X	128.206784	2	1.56E-02
1	188.260577	17	9.03E-02

Supplementary Figure S1. Search of ITS-less alleles in Twilight

Strategy 1: identification of empty alleles in the Trace Database corresponding to ITS-containg alleles in the reference assembly

**Allele in the reference assembly**	ITS
**Alternative allele in Trace Database**	Empty

Identification of ITS loci through BLAST search against the horse reference genome EquCab3.0

↓

For each ITS locus download the sequence from 1 kb upstream to 1 kb downstream

↓

BLAST against the Horse Whole Genome Shotgun sequence Trace Database

↓

Identification of Traces corresponding to empty alleles

Strategy 2: identification of ITS-containing alleles in the Trace Database and of the corresponding empty alleles in the reference assembly

**Allele in the reference assembly**	Empty
**Alternative allele in Trace Database**	ITS

Identification of Traces containing telomeric repeats by BLAST search against Trace Database using sequence $(TTAGGG)_4$ as query

↓

BLAT against EquCab3.0 using each trace sequence as query

↓

Identification of empty alleles in the reference assembly
